# OsKinesin-13A Is an Active Microtubule Depolymerase Involved in Glume Length Regulation via Affecting Cell Elongation

**DOI:** 10.1038/srep09457

**Published:** 2015-03-25

**Authors:** Zhu Yun Deng, Ling Tong Liu, Tang Li, Song Yan, Bai Jian Kuang, Shan Jin Huang, Chang Jie Yan, Tai Wang

**Affiliations:** 1 Key Laboratory of Plant Molecular Physiology, Institute of Botany, Chinese Academy of Sciences, Beijing 100093, China; 2 University of Chinese Academy of Sciences, Beijing 100049, China; 3 Jiangsu Key Laboratory of Crop Genetics and Physiology, Key Laboratory of Plant Functional Genomics of Ministry of Education of China, Agricultural College of Yangzhou University, Jiangsu 225009, China; 4 School of Life Sciences, Tsinghua University, Beijing 100084, China

## Abstract

Grain size is an important trait influencing both the yield and quality of rice and its major determinant is glume size. However, how glume size is regulated remains largely unknown. Here, we report the characterization of OsKinesin-13A, which regulates cell elongation and glume length in rice. The mutant of OsKinesin-13A, *sar1*, displayed length reduction in grains and other organs including internodes, leaves and roots. The grain phenotype in *sar1* was directly caused by reduction in glume length, which in turn restricted caryopsis size. Histological results revealed that length decrease in *sar1* organs resulted from abnormalities in cell elongation. The orientation of cellulose microfibrils was defective in *sar1*. Consistently, *sar1* showed reduced transverse orientation of cortical microtubules. Further observations demonstrated that microtubule turnover was decreased in *sar1*. OsKinesin-13A was shown to be an active microtubule depolymerase and mainly distributed on vesicles derived from the Golgi apparatus and destined for the cell surface. Thus, our results suggest that OsKinesin-13A utilizes its microtubule depolymerization activity to promote microtubule turnover, which may not only influence transverse orientation of cortical microtubules but also facilitate vesicle transport from the Golgi apparatus to the cell surface, and thus affects cellulose microfibril orientation and cell elongation.

Grain size (or shape), usually evaluated by grain length, grain width and grain thickness, is an important quantitative trait for rice because it affects both the yield and the appearance quality of rice. Grain size largely determines grain weight, which is one of the key components of grain yield in rice^1^,^2^. Grain size also decides the dimension and physical appearance of milled white rice, influencing the quality and transaction price of edible rice^3^,^4^. Because rice production contributes significantly to economy and food security, grain size of rice has attracted considerable attention since the emergence of agriculture and has been widely studied and used by rice breeders during the last century^1^. However, it is only in the last decade that scientists began to analyze the control mechanism regulating rice grain size, mainly through identification and functional characterization of genes associated with grain length, width or thickness^1^,^2^,^5^.

Rice grains are composed mainly of outside glumes (lemmas and paleas) and inside caryopses (including embryos, endosperms, and pericarps). The glumes expand their volume during the development of florets and grow to their maximum size before fertilization. Inside the mature glumes, the caryopses start to increase in size after fertilization and reach their final size twenty days after fertilization. Although these two main parts of rice grains grow and develop asynchronously, the final size of caryopses fit perfectly for the inner space enclosed by the glumes. The fact that caryopses grow within glumes after the maturity of glumes makes it reasonable to presume that the dimension of outer glumes may affect the size of inner caryopses and consequently be the main determinant of grain size. A recent study demonstrates that wider glumes promote faster milk filling in caryopsis, leading to widened caryopses and grains in the natural mutants of *GW2*^6^. This result proves glume size is a major factor determining the size of caryopses and grains^7^. However, the mechanism of how glume size influences caryopsis size is still unclear.

Genetic studies of grain size have greatly expanded our knowledge on glume size control in rice, showing that glume size is regulated by multiple genes with pleiotropic and relatively small effects. *SRS1*/*DEP2*, *SRS3*/*OsKINESIN-13A*, *SRS5*, *GL3.1*/*OsPPKL1*, *SG1*, *PGL1*, *APG* and *An-1* mainly affect glume length^8^,^9^,^10^,^11^,^12^,^13^,^14^,^15^. *GW2*, *qSW5*/*GW5*, *GS5*, *GW8*/*OsSPL16* and *HGW* participate in the control of glume width^6^,^7^,^16^,^17^,^18^,^19^. In addition to grain size, the great majority of these genes have pleiotropic effects on other agricultural traits. For example, *SRS1*/*DEP2*, *SRS3*/*OsKINESIN-13A*, *SRS5*, and *SG1* also influence plant height^8^,^9^,^10^,^15^. *HGW*, *GW8*/*OsSPL16* and *An-1* regulate heading date, grain quality and grain number, respectively^11^,^16^,^19^. These glume-size-related genes contribute to grain size regulation through affecting cell number and/or cell size in lemmas and paleas. *GS5*, *GW8*/*OsSPL16* and *GL3.1*/*OsPPKL1* regulate the expression or phosphorylation (for *GL3.1*/*OsPPKL1*) of proteins involved in cell cycle progression, which in turn influences cell division and cell number and consequently affects glume size^12^,^17^,^19^. *GW2* and *qSW5*/*GW5* control the number of cells in glumes by participating in the ubiquitin-proteasome pathway^6^,^7^,^18^. These indicate that glume cell number can be regulated by various molecular pathways. However, the biochemical and molecular mechanisms underlying glume cell-size control remain largely unknown, despite several genes, such as *SRS3*/*OsKINESIN-13A*, *SRS5*, *PGL1* and *APG*, having been identified to influence glume cell length^9^,^10^,^14^. Here, by phenotypic analysis of a new mutant of the *SRS3*/*OsKINESIN-13A* gene, we found that *SRS3*/*OsKINESIN-13A* has a direct effect on glume elongation, which indirectly influences caryopsis enlargement. *SRS3*/*OsKINESIN-13A* plays an essential role in cell elongation in glumes as well as other organs such as internodes, leaves and roots and may regulate cell length through influencing the orientation of cortical microtubules and cellulose microfibrils. The *in vivo* observation of individual microtubule dynamics as well as the *in vitro* biochemical study revealed that OsKinesin-13A is an active microtubule depolymerase involved in the regulation of microtubule dynamics. Localization analysis showed that the OsKinesin-13A protein is mainly localized on Golgi-derived vesicles which destined for the cell surface. Thus, our results showed that OsKinesin-13A may promote cell elongation via its microtubule depolymerizing activity that enhances microtubule turnover and facilitates vesicle transport.

## Results

### Identification of a Rice Round Grain Mutant with Multifaceted Defects

**Figure 1:**
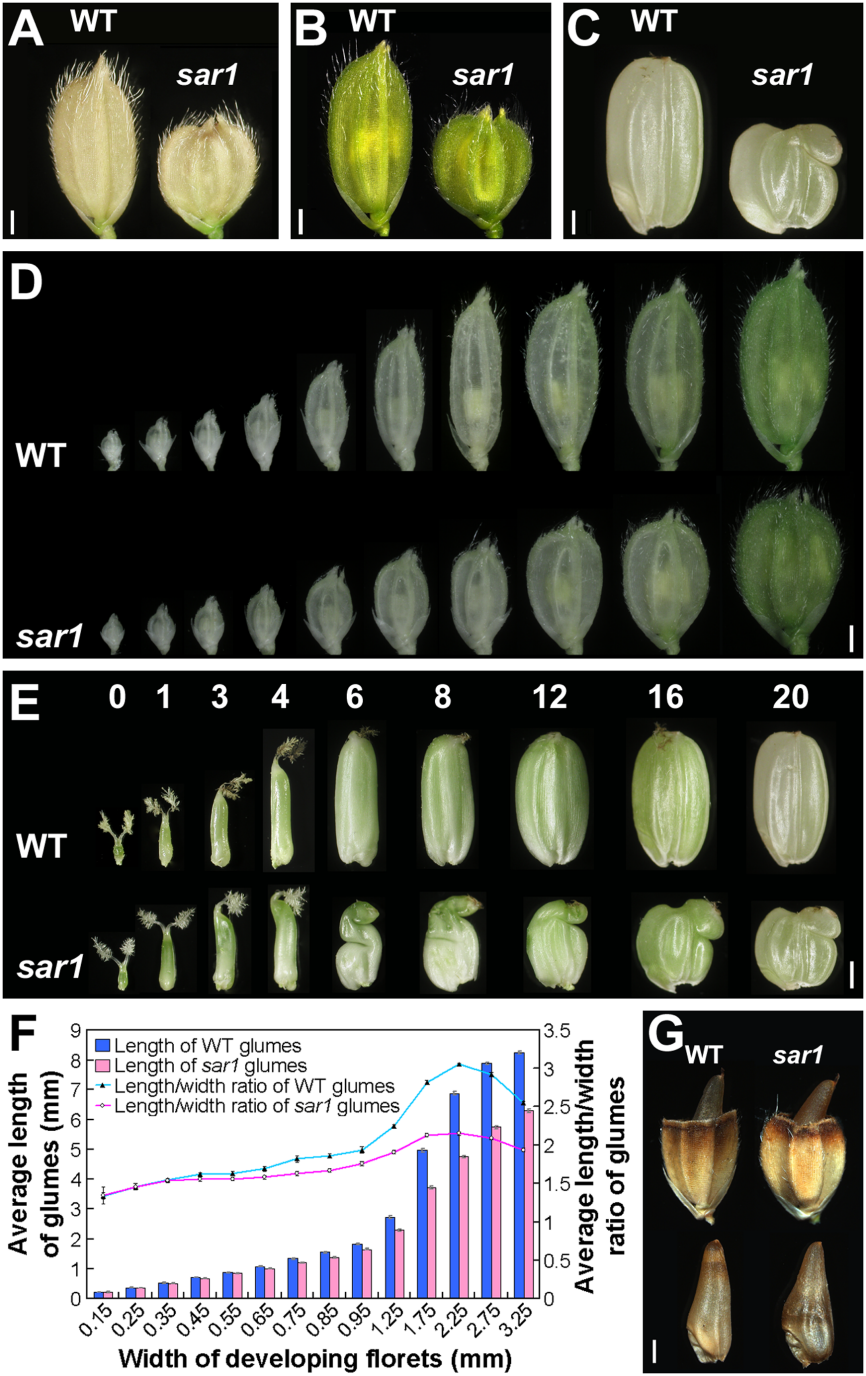
Shortened glumes cause round grains in *sar1*. (A–E) Mature grains (A), mature florets (B), mature caryopses (C), elongating florets (D), developing caryopses (E) of WT and *sar1*. The numbers on Panel E indicate days after pollination. (F) Statistical results of glume size during floral development for the WT and *sar1* (15<n<185). (G) Mature WT and *sar1* grains and caryopses grown under glume-cutting conditions, which mean that the distal ends of glumes were cut off before fertilization, allowing caryopses to develop without height restrictions from glumes. Scale bar = 1 mm in (A) to (E) and (G).

To study the mechanism underlying grain size control in rice, we isolated a γ-ray irradiated mutant producing small and round grains (called *sar1*). Compared with wild-type (WT) control Zhonghua 11, mature *sar1* grains were rounder in shape and lighter in weight (Figure 1A, Table 1). The *sar1* mutant also exhibited size reduction in other organs including internodes, leaves, roots and lodicules, which led to semi-dwarfism and cleistogamy (Supplemental Figure 1 and 2, Supplemental Table 1–3). Furthermore, the fertility of *sar1* was significantly decreased (Supplemental Figure 1I, Supplemental Table 4). Segregation experiments showed *sar1* phenotypes were caused by a single recessive mutation. Positional cloning of *sar1* showed that a single nucleotide deletion within the eighth exon of *SRS3*/*OsKINESIN-13A* resulted in a premature translation termination from the posterior one-third of the kinesin motor domain (Supplemental Figure 3A–D, Supplemental Table 5). Further complementation and RNAi analyses confirmed that this mutation in *SRS3*/*OsKINESIN-13A* is responsible for the *sar1* phenotypes (Supplemental Figure 3E–J, Supplemental Table 4).

### OsKinesin-13A Belongs to a Phylogenetic Subgroup Distinct not only from Animal Kinesin-13s but also from Dicot Kinesin-13As

*SRS3*/*OsKINESIN-13A* encodes a protein belonging to the kinesin-13 family [in the following text, this protein (gene) is called OsKinesin-13A (*OsKINESIN-13A*) to follow the standardized kinesin nomenclature^20^]. To reveal how OsKinesin-13A relates to other kinesin-13 members and provide possible clues to its function, we carried out a detailed phylogenetic analysis of kinesin-13 family members in plants and animals. We retrieved 15 kinesin-13 family members in 6 dicots and 10 members in 4 monocots by BLASTP searches against the NCBI non-redundant database. Phylogenetic analysis of the motor domains of all the 25 plant kinesin-13s and the 14 well-known animal kinesin-13s indicated that plant kinesin-13s were clustered into a large group, which was separate from the two animal kinesin-13 groups, namely Kif2 and Kif24 (Supplemental Figure 4). Plant kinesin-13s were subdivided into Kinesin-13A and Kinesin-13B (Supplemental Figure 4). Interestingly, monocot and dicot Kinesin-13As were sorted into two distinct subgroups. OsKinesin-13A belonged to the monocot subgroup, while AtKinesin-13A, GhKinesin-13A and NtKinesin-13A belonged to the dicot subgroup (Supplemental Figure 4). These results suggest the functional evolution of OsKinesin-13A may have separated it not only from animal kinesin-13 members but also from dicot Kinesin-13A members.

### Mutation in OsKinesin-13A Directly Results in Shortened Glumes, which Indirectly Leads to Shrunken Caryopses

We next analyzed the reason why the grains of *sar1* became round. In *sar1* grains, both glumes and caryopses were greatly reduced in length compared to WT (Figure 1A–C, Table 1). We first observed the elongation process of glumes during floral development and found that the glumes of *sar1* and WT were equal in size (0.22 mm in length) just after finishing the formation of floral organs (0.15 mm in width). However, as the differentiated florets enlarged gradually, the length of *sar1* glumes became shorter than that of WT (Figure 1D and F). Similarly, the length/width ratio of *sar1* glumes was lower than that of WT (Figure 1F). These observations imply that the elongation of glumes is defective in *sar1*. We also observed the dynamic changes in morphology and size of developing caryopses and found that the morphology and size of *sar1* caryopses were similar to that of WT from the 1st to the 3rd day after pollination (DAP) (Figure 1E). However, on the 4th DAP, *sar1* caryopses reached the height of the inner space enclosed by their shortened glumes, and the uppermost part of the *sar1* caryopses bent under the restriction from the reduced space (Figure 1E). During the following 16 days, the developing caryopses of *sar1* exhibited wrinkled and shrunken morphologies (Figure 1E). The wrinkling of *sar1* caryopses varied from a very slight creasing to extreme wrinkling and shrinking. These results indicate that mutation in OsKinesin-13A directly affected the elongation of glumes, thus resulting in shortened glumes. These in turn restrict the development of caryopses, causing wrinkled and compressed caryopses. This notion is supported by the observation that WT and *sar1* caryopses matured under glume-cutting conditions were similar in length (Figure 1G and Table 1).

### OsKinesin-13A Is Involved in Cell Elongation

**Figure 2:**
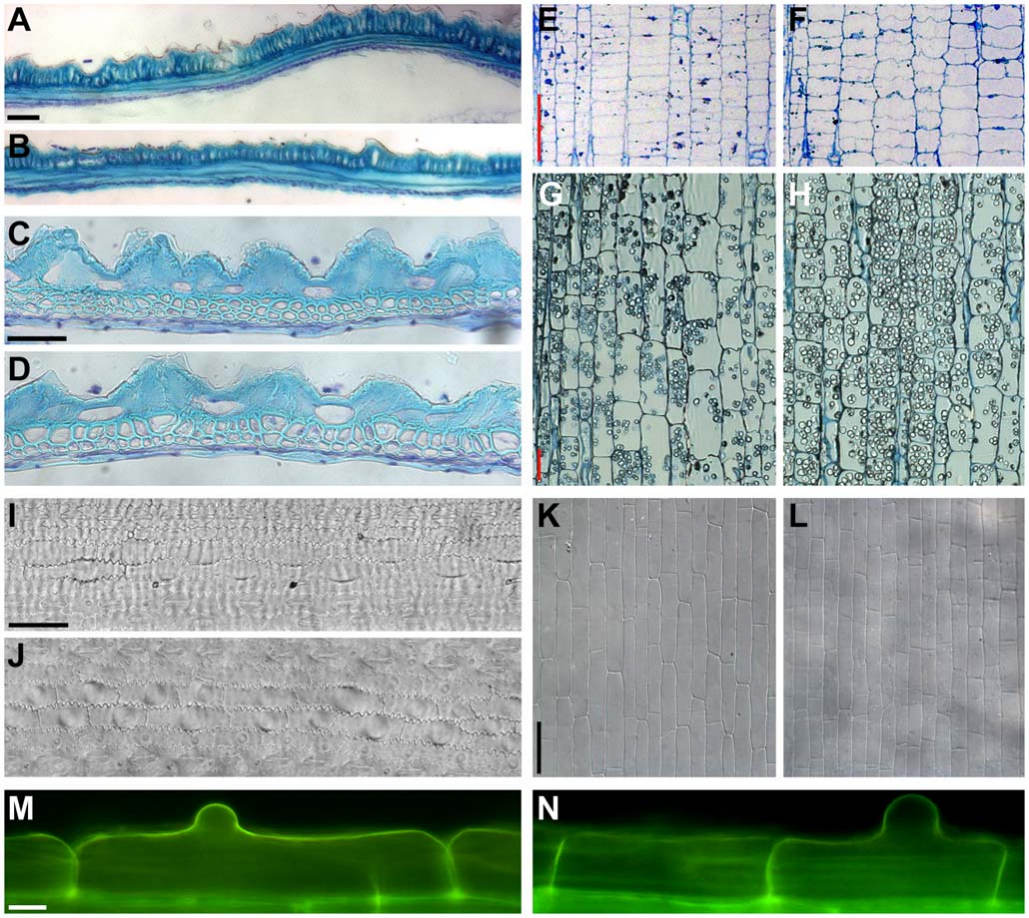
Cell length is reduced in *sar1* glumes, internodes, leaves and roots. (A–D) Longitudinal (A and B) and transverse (C and D) section of the lemma from mature WT (A and C) and *sar1* (B and D) florets. (E–H) Longitudinal section through the intercalary meristem (E and F) and the elongation zone (G and H) of the second internode from mature WT (E and G) and *sar1* (F and H) stems. (I–L) DIC observation of the leaf blade (I and J) and sheath (K and L) from mature WT (I and K) and *sar1* (J and L) flag leaves. (M–N) Fluorescence microscopy of epidermal cells with visible root hair bulges in roots of WT (M) and *sar1* (N). Scale bar = 50 µm in (A) for (A) and (B), in (C) for (C) and (D), in (E) for (E) and (F), in (G) for (G) and (H), in (I) for (I) and (J), in (K) for (K) and (L); bar = 10 µm in (M) for (M) and (N).

We performed cytological observations to analyze the cellular function of OsKinesin-13A in rice glumes. In *sar1*, sclerenchyma cells of mature lemmas were shortened in length (Figure 2A and B, Table 2), but enlarged in area and irregular in shape and size (Figure 2C and D, Table 2) compared to WT. Consistently, cell length, rather than cell number, was also reduced in the inner epidermal cells of lemmas from *srs3*, a published mutant of *OsKINESIN-13A*^9^. These observations indicate mutation in OsKinesin-13A mainly causes defects in cell elongation, thus leading to reduction in glume length.

Given that *sar1* had shortened internodes, we then used internodes as model systems to further assess whether mutation in OsKinesin-13A only influenced cell elongation. In this regard, the internode has the advantage that cell division and elongation occur in its different regions, namely the intercalary meristem and the elongation zone, respectively^21^. In longitudinal sections, *sar1* dividing cells in the intercalary meristem were small and tightly packed, morphologically analogous to WT (Figure 2E and F). However, *sar1* parenchymal cells at the top of the elongation zone, representing cells that had finished elongation and reached their final length, were shorter than WT (Figure 2G and H, Table 2). These results indicate that mutation in OsKinesin-13A affects cell elongation rather than cell division. Similarly, the defective cell elongation phenotype was also observed in *sar1* cells from other organs, such as epidermal cells in flag leaf blades and sheaths (Figure 2I–L, Table 2), and root epidermal cells with visible root hair bulges (which represent cells at the end of elongation) (Figure 2M and N, Table 2). Taken together, these data imply that OsKinesin-13A is involved in organ size control via regulating cell elongation.

### OsKinesin-13A Has an Effect on Cellulose Microfibril Orientation

**Figure 3:**
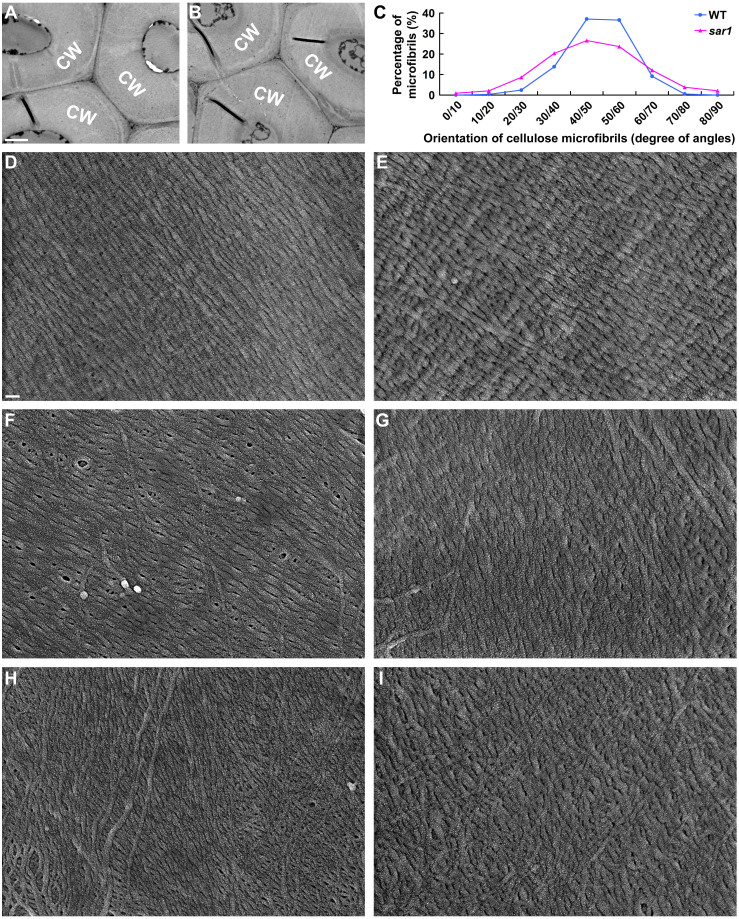
Sclerenchyma cells of *sar1* show abnormalities in the orientation of cellulose microfibrils in secondary cell walls. (A–B) Transmission electron micrographs of the sclerenchyma cell walls (CW) of WT (A) and *sar1* (B) stems. (C) Quantitative analysis of cellulose microfibril orientation in cells where microfibrils were neatly aligned parallel to each other. Sclerenchyma cells in the stem elongation zone showed three different types of cellulose microfibril arrays, exemplified in D and E, F and G, H and I, respectively. Representative images of cell walls from WT and *sar1* cells (4–5 cells from 3 different plants) with microfibril arrays as those in D (WT) and E (*sar1*) were used to measure microfibril orientation. The direction transverse to the elongation axis is defined as zero degree. The horizontal axis shows the orientation of cellulose microfibrils divided into groups, e.g. 0/10 indicates the orientation ranges from more than 0 degree to less than or equal to 10 degree in this group. (D–I) Scanning electron micrographs of cellulose microfibrils in the innermost layer of WT (D, F, H) and *sar1* (E, G, I) sclerenchyma cell walls. Scale bars = 1 µm in (A) for (A) and (B), in (D) for (D) to (I).

For a plant cell, its size and growth direction are determined by its wall^22^. Thus, we tested whether the defective cell elongation in *sar1* resulted from morphological or thickness abnormities in cell walls. Observation of the sclerenchyma cells in the stem elongation zone showed that WT and *sar1* had no obvious differences in wall morphology (Figure 3A and B) and thickness (1.95 ± 0.31 vs. 1.92 ± 0.41 μm, *P* value of Student's t-test = 0.6).

In addition, the arrangement pattern of cellulose microfibrils, which is the basic structural backbone of cell walls, is supposed to play a key role in the determination of growth direction^23^. Therefore, we examined the arrangement of the cellulose microfibrils in *sar1* and WT cells by visualizing the innermost secondary walls of sclerenchyma cells in the stem elongation zone with a field emission scanning electron microscope. We observed three types of cellulose microfibril arrays in WT. In individual sclerenchyma cells from the basal portion of the stem elongation zone, their cellulose microfibrils showed totally parallel array, which meant almost all of the cellulose microfibrils in the same cell were neatly aligned (Figure 3D). In sclerenchyma cells from the middle portion of the stem elongation zone, cellulose microfibrils remained predominantly aligned in parallel, although a small number of microfibrils deviated obviously from the main direction (Figure 3F). In sclerenchyma cells from the upper portion of the stem elongation zone, cellulose microfibrils were randomly oriented (Figure 3H). The three types of cellulose microfibril arrays were also present in *sar1* (Figure 3E, G and I), but *sar1* had obvious defects in the arrangement of microfibrils. Firstly, the stem elongation zone of *sar1* contained lower proportion of cells with the totally parallel array than that of WT (4.3% in *sar1* vs. 29.1% in WT). Secondly, cellulose microfibrils of WT cells with the totally parallel array were prominently oriented in the direction that makes an angle of 40–60° with the transverse axis; however, the angles of cellulose microfibrils in *sar1* cells with such arrays had a slightly wider distribution (Figure 3C). These observations suggest that OsKinesin-13A may participate in cell elongation through affecting the orientation of cellulose microfibrils in cell walls.

### OsKinesin-13A Influences the Transverse Orientation of Cortical Microtubules

**Figure 4:**
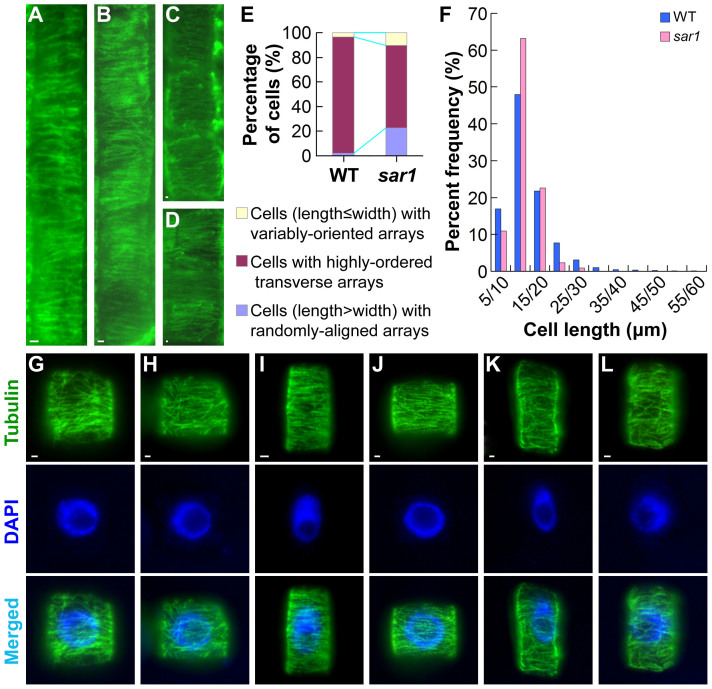
*sar1* shows reduced transverse orientation of cortical microtubules. (A–D) Immunofluorescence of microtubules in the elongating epidermal (A and B) or parenchyma (C and D) cells of WT (A and C) and *sar1* (B and D) stems. (E) Percentage of root cells at the early, rapid and late elongation stages that have microtubule arrays exemplified in (G and H), (I and J) and (K and L), respectively (n = 877 and 517 cells from 10 different plants for WT and *sar1*, respectively). (F) Percent frequency distribution of root cells with highly-ordered transverse microtubule arrays (n = 831 and 345 cells from 10 different plants for WT and *sar1*, respectively). The horizontal axis shows the cell length divided into groups, e.g. 5/10 indicates the cell length ranges from more than 5 µm to less than or equal to 10 µm in this group. (G–L) Immunofluorescence of microtubules in cells at the root elongation zone of WT (G, I and K) and *sar1* (H, J and L). Bar = 1 µm in (A–D) and (G–L).

The orientation of cortical microtubules is a major determinant of the arrangement of cellulose microfibrils^24^,^25^, we therefore compared cortical microtubule arrays of WT and *sar1* cells in the stem and root elongation zone by immunofluorescence staining with anti-tubulin antibodies. In elongating epidermal (Figure 4A) and parenchyma (Figure 4C) cells of WT stems, cortical microtubules were well organized and highly aligned in transverse arrays. Such transverse microtubule arrays were also observed in elongating cells of *sar1* stems (Figure 4B and D). Detailed observations of root elongating cells showed that as WT (Figure 4G, I and K), *sar1* was able to form three types of microtubule arrays, namely the variably-oriented array in cells with length (along the longitudinal axis) shorter than or equal to width (along the transverse axis) (Figure 4H), the highly-ordered transverse array (which meant at least 95% of the microtubules in a given cell were oriented in the direction that makes an angle of −30°–30° with the transverse axis) (Figure 4J), and the randomly-aligned array in cells with length longer than width (Figure 4L). By comparison, *sar1* contained a lower percentage of cells with the highly-ordered transverse arrays than WT (Figure 4E). Compared to WT cells with the transverse arrays, *sar1* cells with such arrays were distributed in a narrower length range, concentrating mainly in 10–20 μm but missing from 30–60 μm (Figure 4F). These results showed that mutation in OsKinesin-13A influenced the transverse orientation of cortical microtubules, which, to some extent, explains the defects in the orientation of cellulose microfibrils in *sar1*.

### OsKinesin-13A Promotes the Turnover of Microtubules

**Figure 5:**
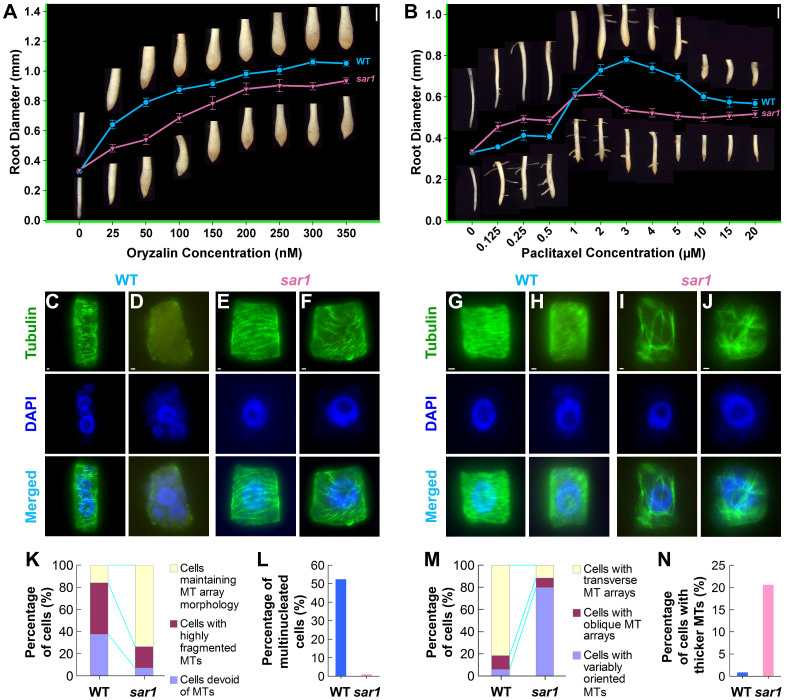
*sar1* differentially responds to anti-microtubular drugs. (A) *sar1* is more resistant to microtubule depolymerizing drug oryzalin. (B) *sar1* is more sensitive to microtubule stabilizing drug paclitaxel. Values of solid curves show the mean root tip diameters of WT (closed cycles, blue line) and *sar1* (inverted triangles, red line) at each treated concentration. Error bars show the standard errors of the mean from triplicate experiments. Light stereomicrographes of root tips from WT and *sar1* seedlings treated with anti-microtubular drugs are shown on the upper and lower sides of the curves, respectively. The bars are 1 mm, represented by the white vertical lines on the upper right corners of (A) and (B). (C–J) Microtubule (MT) phenotypes in elongating root cells of WT (C, D, G and H) and *sar1* (E, F, I and J) after 250 nM oryzalin (C–F) or 25 μM paclitaxel (G–J) treatment. Bar = 1 µm in (C) to (J). (K–N) Quantitative analysis of microtubule phenotypes caused by oryzalin (K and L) or paclitaxel (M and N) treatment; n = 1117–1556 cells from 6–8 different plants.

Given that microtubule turnover is important for the orientation of cortical microtubules^26^,^27^ and OsKinesin-13A belongs to a family of proteins involved in the regulation of microtubule dynamics^28^,^29^, we tested whether loss of OsKinesin-13A affect microtubule turnover through anti-microtubular drug sensitivity assay as well as direct visualization of individual microtubule dynamics. First, we treated germinated seedlings with the microtubule depolymerizing drug oryzalin and the microtubule stabilizing drug paclitaxel and tested whether *sar1* differs from WT in drug tolerance by analyzing root swelling. After oryzalin treatment, *sar1* roots showed a morphological change similar to WT roots, but the extent of root swelling decreased at each treated concentration (Figure 5A). The *sar1* roots responded to paclitaxel treatment in a pattern similar to WT (Figure 5B). However, the extent of root swelling was greater in *sar1* than in WT at lower paclitaxel concentration (0–1 µM) (Figure 5B). Furthermore, *sar1* roots reached their maximum enlargement and began to stop their radial growth at concentrations lower than those required for WT roots (Figure 5B). These data indicate that *sar1* is more tolerant to drugs that promote microtubule depolymerization but more sensitive to drugs that stabilize microtubules.

We next visualized directly the effect of drug treatment on microtubule organization. After treatment with 250 nM oryzalin or 25 μM paclitaxel, microtubule arrays in elongating root cells of *sar1* and WT were compared. Oryzalin treatment led to highly fragmented microtubules that lost their normal transverse alignment (Figure 5C) or almost complete absence of microtubules (Figure 5D) in most of the WT elongating cells (Figure 5K). However, most of the *sar1* elongating cells maintained the morphology of their microtubule arrays after oryzalin treatment, although fragmentation was present on microtubules (Figure 5E, F and K). In addition, more than half of the WT root cells became multinucleated after oryzalin treatment (Figure 5C, D and L), but multinucleation is hardly observed in oryzalin-treated *sar1* roots (Figure 5E, F and L). In WT roots, paclitaxel incubation resulted in an increase in the intensity and density of microtubules without altering the transverse orientation of microtubules in most elongating cells (Figure 5G and M), and obliquely-aligned microtubules in a small proportion of elongating cells (Figure 5H and M). In contrast, most elongating cells from the paclitaxel-treated *sar1* roots had variably-oriented microtubule arrays (Figure 5I, J and M) and part of *sar1* cells showed thicker microtubules with brighter fluorescent signals (Figure 5J and N). These observations showed that compared to WT, *sar1* had a less severe microtubule phenotype after oryzalin treatment but a more severe microtubule phenotype after paclitaxel treatment, which is consistent with the changes in tolerance to anti-microtubular drugs in *sar1*.

**Figure 6:**
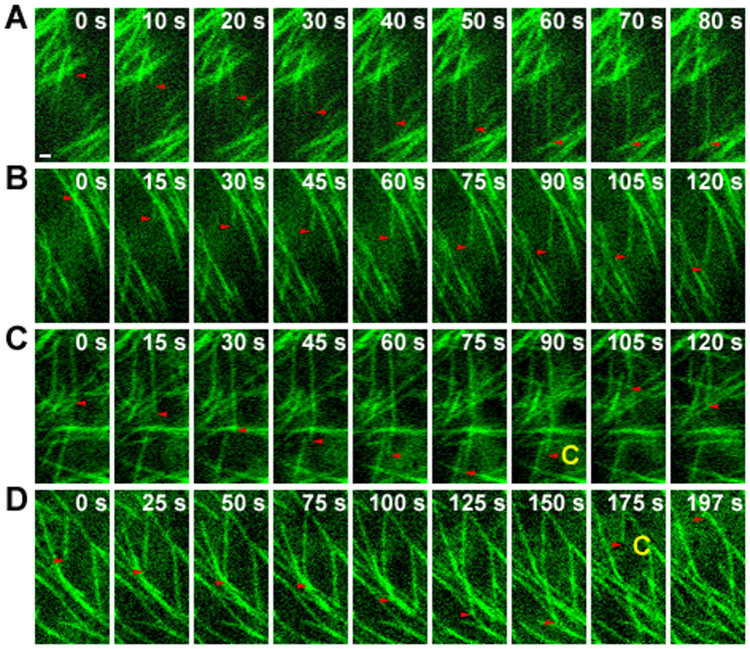
The dynamic instability behavior of individual microtubules is altered in *sar1*. (A–D) Sequential images from time-lapse movies of WT (A and C) and *sar1* (B and D) cells expressing EGFP-tagged α-tubulin. Red arrowheads track the plus ends of selected microtubules. White numbers indicate the elapsed time (seconds). Yellow letter C highlights microtubule catastrophe events (switches from growth to shrinkage). Scale bar = 1 µm in (A) for (A) to (D). See the entire series in Supplemental Movie 1 to 4 that correspond to panel A to D, respectively.

To confirm directly that OsKinesin-13A regulates the turnover of microtubules *in vivo*, we analyzed the dynamic instability behavior of individual microtubules in WT and *sar1* cells that expressing enhanced green fluorescent protein (EGFP)-tagged α-tubulin. In *sar1* root cells (Figure 6B and D, Table 3, Supplemental Movie 2 and 4), the microtubule growth rate and catastrophe (the switch from growth to shrinkage) frequency were significantly reduced compared to that in WT (Figure 6A and C, Supplemental Movie 1 and 3). Microtubules in *sar1* spent less time in the shrinkage phase but more time in the pause phase (Table 3). Moreover, *sar1* displayed an increase in the microtubule shrinkage rate (Table 3). The microtubule rescue (the switch from shrinkage to growth) frequency and the percent of time that microtubules spent in the growth phase seemed not influenced in *sar1* (Table 3). Overall, microtubules of *sar1* had lower dynamicity than those of WT (Table 3). The changes in the parameters of microtubule dynamic instability in *sar1* showed OsKinesin-13A may promote microtubule turnover mainly by stimulating microtubule catastrophe.

### OsKinesin-13A Has a Microtubule Depolymerization Activity *in Vitro*

Animal kinesin-13s can efficiently couple ATP hydrolysis to microtubule depolymerization, and they use these activities to regulate microtubule dynamics in mitotic and interphase cells^29^,^30^,^31^. To determine whether OsKinesin-13A utilizes a similar molecular mechanism to regulate the turnover of cortical microtubules, the His-tagged OsKinesin-13A motor domain (OsKinesin-13A-motor) and the untagged full-length OsKinesin-13A protein (full-length OsKinesin-13A) were expressed in *E.coli* and purified for assaying the ATPase, microtubule-depolymerization and tubulin-binding activities (Supplemental Figure 5).

**Figure 7:**
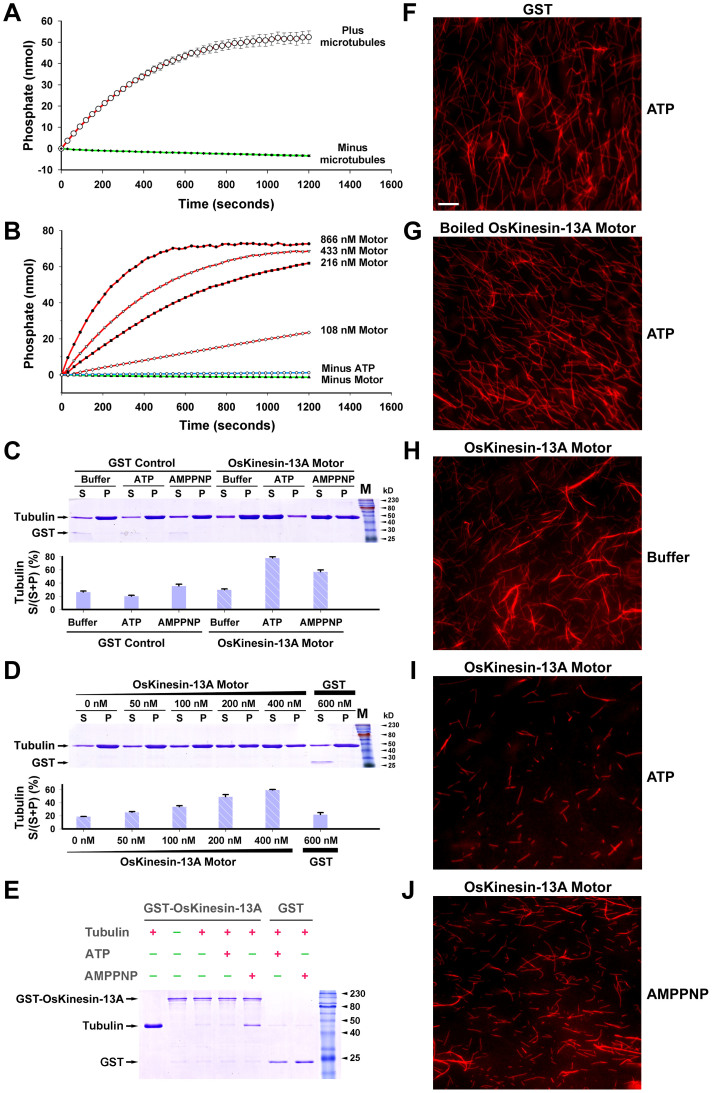
Motor domain of OsKinesin-13A depolymerizes microtubules *in vitro*. (A) and (B) Microtubule-dependent ATPase activity of OsKinesin-13A-motor. Each point on the curves represents the mean (nanomoles, nmol) and standard errors (error bars) for Pi release as determined in triplicate by ELIPA. (C) and (D) Sedimentation analysis of microtubule depolymerization induced by OsKinesin-13A-motor. Microtubule depolymerization reaction was performed with GST Control or OsKinesin-13A-Motor (1.6 μM in C) in the absence (Buffer) or presence of ATP or AMPPNP. Reaction mixtures were centrifuged to separate supernatants (S) and pellets (P), which were analyzed by SDS-PAGE. The stained SDS-PAGE gel is shown in the upper panels. The bands of GST and tubulin are indicated on the left. The protein bands of OsKinesin-13A-motor are invisible in gels because the recombinant motor protein (48 kD) has a molecular weight close to tubulins (55 kD). The lower panels show the statistical results from three independent experiments. The mean percentage of tubulin in the supernatant versus supernatant and pellet [tubulin S/(S + P)%] ± standard errors of the mean (error bars) are shown. (E) Tubulin heterodimers cosediment with OsKinesin-13A. Beads bound to GST-OsKinesin-13A or GST were used in tubulin binding assay in the presence (red plus sign) or absence (green minus sign) of ATP, AMPPNP and tubulin heterodimers. (F–J) Visual assay of microtubule depolymerization by OsKinesin-13A-motor. Bar = 10 µm in (F) for (F) to (J).

The ATPase and depolymerization activities of kinesin-13s were conferred by their conserved kinesin motor domain^32^. Therefore, OsKinesin-13A-motor was first used to determine whether OsKinesin-13A has these biochemical activities. The Enzyme Linked Inorganic Phosphate Assay (ELIPA) showed that OsKinesin-13A-motor can catalyze the hydrolysis of ATP to ADP and phosphate (Pi) in the presence of microtubules and increasing concentrations of OsKinesin-13A-motor led to progressive increase in Pi release (Figure 7A and B), indicating that OsKinesin-13A is an active ATPase.

The microtubule depolymerization assays demonstrated that when paclitaxel-stabilized microtubules were incubated with OsKinesin-13A-motor in the presence of ATP or its nonhydrolyzable analogue AMPPNP, the disassembly of microtubule polymers was greatly increased (Figure 7C, I and J). Increasing the concentration of OsKinesin-13A-motor caused increased depolymerization of microtubules (Figure 7D). As negative controls, the glutathione S-transferase (GST) protein or boiled OsKinesin-13A-motor had no depolymerizing activity (Figure 7C, D, F and G). These results showed that OsKinesin-13A depolymerizes paclitaxel-stabilized microtubules in a concentration-dependent manner, for which ATP hydrolysis is not essential. Furthermore, full-length OsKinesin-13A exhibited a higher level of microtubule depolymerization activity compared to OsKinesin-13A-motor (Supplemental Figure 6). This observation indicates although the microtubule depolymerization activity of OsKinesin-13A is defined by its motor domain, protein sequences outside the motor domain also contribute to its depolymerization activity.

Biochemical studies in animals have demonstrated that ATP hydrolysis is required for dissociation of kinesin-13 proteins from tubulin heterodimers, although it is not essential for the release of tubulin heterodimers from microtubule polymers^33^,^34^. To confirm whether OsKinesin-13A uses the same mechanism to couple ATP hydrolysis to microtubule depolymerization, we performed tubulin binding assay with the GST-tagged full-length OsKinesin-13A protein (GST-OsKinesin-13A). As a control, the GST itself did not interact with tubulin dimers in the presence of either ATP or AMPPNP (Figure 7E). GST-OsKinesin-13A was pulled down with tubulin dimers in the presence of AMPPNP (Figure 7E). However, when ATP was present, GST-OsKinesin-13A was not associated with tubulin dimers. This observation proves that ATP hydrolysis is necessary for the dissociation between OsKinesin-13A and tubulin heterodimers.

### OsKinesin-13A Is Mainly Localized on Vesicles

The localization of a protein correlates strongly with its cellular and molecular functions. The previous study has showed that OsKinesin-13A is present in the total membrane fraction^9^. We further separated crude microsomal membranes into organelle membrane-enriched fractions by sucrose density gradient centrifugation and analyzed the distribution of OsKinesin-13A across these fractions by Western blotting with a polyclonal antibody specific for OsKinesin-13A (Supplemental Figure 7). OsKinesin-13A was more abundant in the fractions where the marker proteins for the plasma membrane and Golgi apparatus were peaked (Supplemental Figure 8), suggesting OsKinesin-13A may be connected with the two organelles.

**Figure 8:**
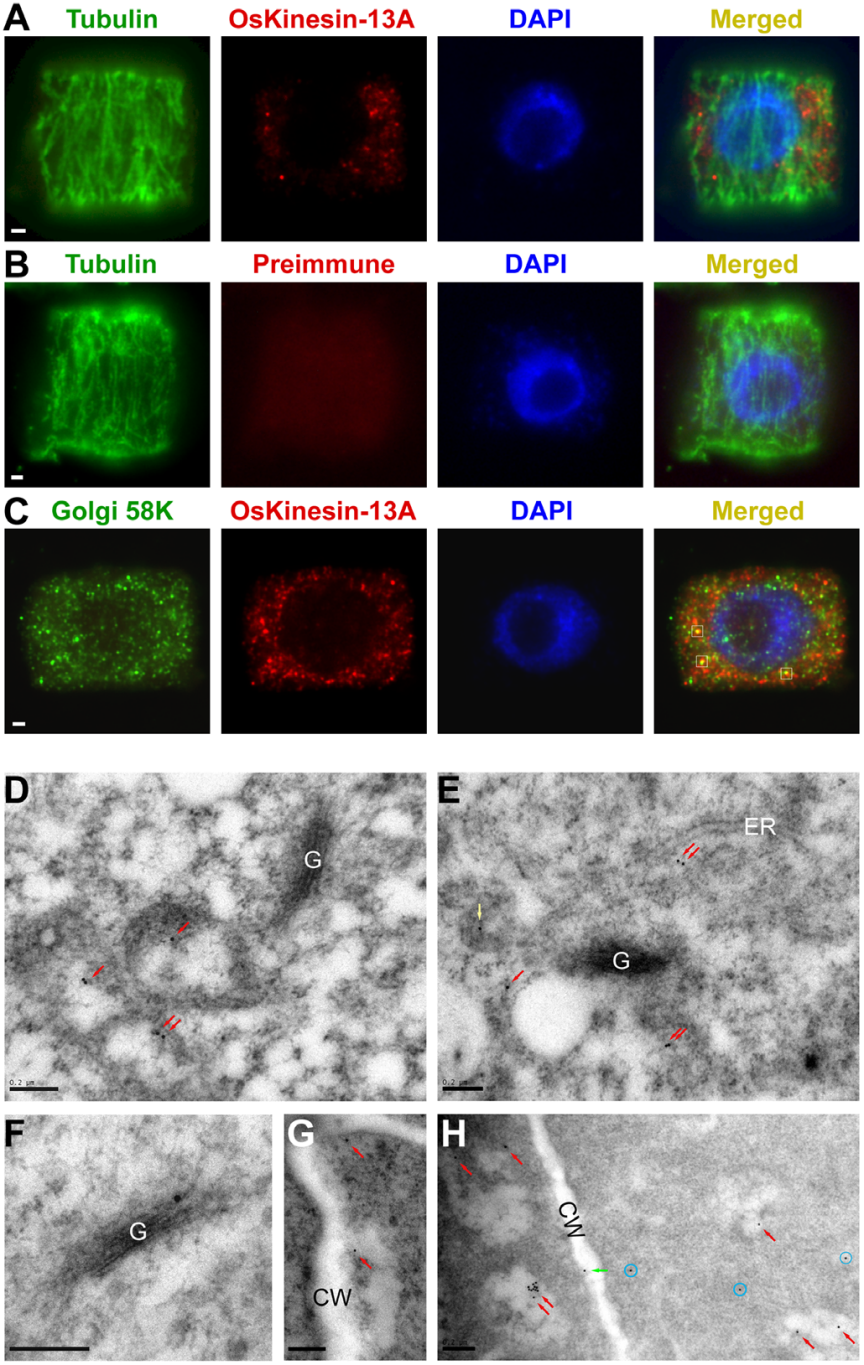
Localization of OsKinesin-13A in cells. (A–C) Subcellular localization of OsKinesin-13A in cells. WT root tip cells were hybridized with antibodies against the proteins indicated on micrographs. The preimmune rabbit serum was used as a negative control and is indicated by preimmume. The nuclei were counterstained with DAPI (blue). White squares in C indicate the sites where OsKinesin-13A signals (red) overlapped with 58K signals (green). (D–H) Ultrastructural localization of OsKinesin-13A in root tip cells. The red, green, yellow arrows point to gold particles on vesicles, cell membranes and endoplasmic reticulums, respectively. The gold particles dispersed in the cytoplasm are indicated by blue circles. G, Golgi stacks; CW, cell walls; ER, endoplasmic reticulums. Bar = 1 μm in (A) to (C) and 200 nm in (D) to (H).

Furthermore, we performed immunofluorescence and immunogold analyses to determine the localization of OsKinesin-13A in cells. The immunofluorescence result showed that OsKinesin-13A displayed a punctate distribution throughout the cytoplasm in different kinds of cells (Figure 8A and Supplemental Figure 9). Dicot Kinesin-13A members such as AtKinesin-13A, GhKinesin-13A and NtKinesin-13A are localized to the Golgi apparatus, which also shows as punctate signals^35^,^36^,^37^. To confirm whether OsKinesin-13A is associated with the Golgi apparatus as dicot Kinesin-13As, we labeled the Golgi apparatus with an antibody against the Golgi marker 58K^37^. Double-label immunofluorescence results showed no obvious colocalization of OsKinesin-13A with this Golgi marker, although a very small number of OsKinesin-13A signals overlapped with 58K signals (Figure 8C).

The immunogold electron microscopic study showed that more than half of the OsKinesin-13A labeling was observed on vesicle-like structures, which were often in proximity to Golgi stacks or cell membranes (Figure 8D, E and H, Table 4). Some of the labeled vesicles were fused with the cell membranes (Figure 8G). Approximately one-third of OsKinesin-13A labeling was found in the cytoplasm (Figure 8H, Table 4). In addition, a small number of OsKinesin-13A labeling was distributed in cell membranes, Golgi stacks and endoplasmic reticulums (Figure 8H, F and E, respectively, Table 4). These results suggest that OsKinesin-13A is mainly localized on vesicles rather than Golgi stacks, but the OsKinesin-13A-labeled vesicles may be derived from Golgi stacks and delivered to cell membranes.

## Discussion

### Regulation of Caryopsis Size by Glume size in rice

Previous study has demonstrated that OsKinesin-13A deficiency causes small and round grains^9^. However, it remains unclear whether the phenotype results from the defect in glumes or caryopses or both. Our study further revealed that although the two main parts of grains, namely glumes and caryopses, were both diminished in length in *sar1*, mutation in OsKinesin-13A only directly affected glume elongation and glume length. The length reduction in *sar1* caryopses was not a direct consequence of mutation in OsKinesin-13A, but resulted from the space restrictions imposed by the shortened glumes. These results indicate that OsKinesin-13A is a gene involved in determination of glume size rather than caryopsis size. The observation that *sar1* caryopses grew to the length equal to WT after removal of the height constraints imposed by glumes suggests that caryopsis size may be initially determined by genetic factors other than OsKinesin-13A and can be subsequently influenced by the size of glumes. Actually, the outer glumes provide an effective protection for the inner caryopses under normal circumstances, ensuring the caryopses develop normally and expand to their determinate size. However, the outer glumes would restrict the growth of the inner caryopses if the glumes reduce in size for a certain reason. In line with this notion, the WT caryopses developed in top-half-cut glumes were unable to grow to the normal shape and size; their length, width, and thickness were decreased compared with those developed in intact glumes (Figure 1G, Table 1). On the contrary, the *sar1* caryopses were increased in length when the apexes of their glumes were removed (Figure 1G, Table1). Further investigations are necessary to elucidate how the developing caryopsis communicates with their developed glumes to coordinate its growth with the size of glumes.

### Plant and Animal Kinesin-13s Have Conserved Biochemical Activities but Diverse Cellular Functions

Animal kinesin-13 family members are well-known microtubule depolymerases^30^,^38^,^39^. Recently, a study of AtKinesin-13A shows this dicot kinesin-13 member is necessary for depolymerization of cortical microtubules^40^. Here, our results provide conclusive evidence that monocot kinesin-13s can depolymerize microtubules *in vitro* and *in vivo*, suggesting that the microtubule depolymerization activity of kinesin-13s is well conserved in plants. As shown by the biochemical assays, both the full-length OsKinesin-13A proteins and its motor domain were able to catalyze the depolymerization of paclitaxel-stabilized microtubules *in vitro*. Furthermore, mutation in OsKinesin-13A reduced the severity of microtubule abnormalities induced by treatment with the microtubule depolymerizing drug but increased the severity of microtubule abnormalities induced by the microtubule stabilizing drug. Mutation in OsKinesin-13A also changed the dynamic instability behavior of cortical microtubules, such as the reduction in the microtubule growth rate and catastrophe frequency. These *in vivo* results confirm that OsKinesin-13A is an active microtubule depolymerase required for promoting the turnover of microtubules *in vivo*. For animal kinesin-13s, an important characteristic of their depolymerization mechanism is that they use their ATP-hydrolysis activities to dissociate themselves from tubulin dimers^33^,^34^. This feature is also shared by plant kinesin-13s because OsKinesin-13A is associated with tubulin dimers in the presence of the nonhydrolyzable ATP analogue AMPPNP but dissociated from tubulin dimers in the presence of ATP (Figure 7E).

Although plant and animal kinesin-13s share the conserved biochemical activities, they are divergent in cellular functions. The most important function of animal kinesin-13 members is to participate in microtubule-dependent cellular processes during mitosis, e.g. spindle assembly, spindle bipolarity establishment, and chromosome segregation^29^,^30^,^33^. In contrast, mutation in AtKinesin-13A^35^ or OsKinesin-13A (Ref. 9 and this study) did not induce defects in mitosis, which implies that plant kinesin-13 members may not be necessary for mitosis. Consistent with the results from genetic studies, our phylogenetic analysis demonstrated that plant and animal kinesin-13 members were sorted into two discrete groups, suggesting functional evolution of plant and animal kinesin-13s are separate from each other.

It is interesting that on the phylogenetic tree, monocot Kinesin-13A proteins clustered together rather than dispersed into the dicot Kinesin-13A group, indicating monocot Kinesin-13A members might be functionally different from their dicot homologs. Evidences to support this claim are provided by the following observations. Firstly, although both *AtKINESIN-13A*^36^ and *OsKINESIN-13A*^9^ are expressed in various organs such as roots, stems, and leaves, deficiency of AtKINESIN-13A only leads to increased branches in leaf trichomes^35^ and smaller secondary cell wall pits in xylem cells^40^. In contrast, mutants of the *OsKINESIN-13A* gene displayed multifaceted defects in leaves, stems, roots, florets and panicles, and grains (Ref. 9 and this study). These observations imply that in most tissues and cells, the functions of AtKinesin-13A may overlap significantly with that of other kinesins, AtKinesin-13B in particular. By contrast, OsKinesin-13A may evolve to have distinct functions. Furthermore, OsKinesin-13A plays a general role in regulating cell elongation, since reduction in cell length was observed in glumes of *sar1* and *srs3*, and internodes, leaves and roots of *sar1* (Ref. 9 and this study). However, AtKinesin-13A plays specialized roles in Golgi organization required for trichome morphogenesis^35^, in budding of Golgi-associated vesicles in root-cap peripheral cells^36^ and in formation of secondary wall pits in xylem cells^40^.

### Possible Mechanisms connecting microtubule depolymerization and cell elongation

Our biochemical and cellular studies showed that OsKinesin-13A is a microtubule depolymerase involved in cell elongation. One question raised by this result is what molecular mechanism OsKinesin-13A uses to connect its microtubule depolymerization activity with its function in cell elongation.

In plants, cell elongation requires cell wall expansion, which depends mainly on the massive deposition of newly-synthesized wall components to the old and loosened wall^41^,^42^. The major load-bearing component of the cell wall, cellulose, is synthesized on the cell membrane by cellulose synthase complexes^43^. It is well known that microtubules are involved in cell elongation by influencing the deposition of cellulose microfibrils in cell walls. During cell elongation, cortical microtubules form transversely aligned arrays, which guide the movement of cellulose synthases in the plasma membrane and consequently allow cellulose microfibrils to deposit transversely and orderly on expanding cell walls^25^,^42^,^44^,^45^,^46^. Such transversely-oriented cellulose microfibrils restrict cells to expanding mainly in the longitudinal direction. Several microtubule-associated proteins such as SKU6/SPIRAL1^47^, MAP18^48^, MDP40^49^ have been identified to use this molecular mechanism to participate in cell elongation. Mutation in any of these proteins causes disrupted microtubule arrays, which induced disordered cellulose microfibrils and consequently shortened cells. Our results showed *sar1* elongating cells can generate all types of normal microtubule arrays, but had a reduced proportion of ordered transverse arrays. Consistently, the elongation zone of *sar1* had a decreased proportion of cells with totally parallel arrays of cellulose microfibrils and the orientation of cellulose microfibrils in such cells were more widely distributed than that in WT. Thus, OsKinesin-13A, a microtubule depolymerase and regulator of microtubule dynamic instability, may influence cellulose microfibril orientation and cell elongation through regulating the transverse orientation of cortical microtubules that guide the movement of cellulose synthases beneath the plasma membrane.

Although cellulose microfibrils are formed on the cell membrane, subunits of the protein complex responsible for their synthesis, namely cellulose synthases, are synthesized and assembled in the endoplasmic reticulum^50^. The assembled cellulose synthase complexes are transferred to the Golgi stacks, from which they are inserted into the plasma membrane directly^43^,^50^ or after delivery to a kind of secretory vesicle named microtubule-associated cellulose synthase compartment (MASC)^51^ or small cellulose synthase compartment (SmaCC)^52^. In contrast to cellulose, non-cellulose polysaccharides in wall matrix, such as pectin, are synthesized in Golgi stacks and transported to cell membrane by secretory vesicles. Our localization results showed that OsKinesin-13A was mainly distributed on vesicles, and several pieces of evidence indicate that these OsKinesin-13A-labeled vesicles may be involved in delivery of materials related to the synthesis of cell wall components. Firstly, OsKinesin-13A was relatively abundant in the membrane fractions enriched with the plasma membrane and the Golgi apparatus. Secondly, most of the OsKinesin-13A-labeled vesicles were located in the vicinity of cell surface or the Golgi apparatus and some of them were in the stage of fusion with the plasma membrane. These results suggest the OsKinesin-13A-labeled vesicles may be derived from the Golgi apparatus and destined for cell surface, just like the secretory vesicles delivering cellulose synthase complexes or non-cellulose wall polysaccharides. Thirdly, similar to secretory vesicles mediating the transport of wall polysaccharides from Golgi to cell surface^53^, the OsKinesin-13A-labeled vesicles are highly variable in size (diameter ranging from 100 to 800 nm). Co-localization experiments using markers specific for different wall materials will determine what kind of materials the OsKinesin-13A-labeled vesicles transfer from the Golgi apparatus to cell surface.

Cortical microtubules have been detected to co-localize with secretory vesicles such as MASC/SmaCC involved in the secretion of cellulose synthase complexes^51^,^52^ and electron-dense vesicles required for pectin secretion in seed coat cells^54^. Cellular and molecular studies further showed that MASC/SmaCC can track the plus or minus ends of microtubules and their movement is driven by microtubule depolymerization^51^,^52^. Although it is still unclear whether microtubule end-tracking kinesins are involved in MASC/SmaCC tethering to microtubules^52^, kinesin-13 family members are good candidates for connecting MASC/SmaCC with microtubule ends because kinesin-13s can target and depolymerize both ends of microtubules^30^,^38^. It will be interesting to determine whether OsKinesin-13A uses its microtubule binding and depolymerization activities to regulate the mobility of secretory vesicles serving to traffic wall materials and whether OsKinesin-13A contributes to cellulose microfibril orientation not only by regulating transverse orientation of cortical microtubules but also by influencing vesicle transport from the Golgi apparatus to the cell surface.

## Methods

### Plant Materials and Growth Conditions

Rice WT cultivar Zhonghua 11 (*Oryza sativa* *japonica*) was used to generate populations of mutant lines. WT cultivar Nanjing 11 (*O. sativa indica*) was used for genetic analysis and positional cloning. All WT, mutant, and F_2_ rice plants were grown in paddy fields under natural conditions during the summer growing season.

### Cytological Analysis and Microscopy

For histological analysis, mature florets or lemmas were fixed in FAA, dehydrated with a graded tert-butyl alcohol series, embedded in Paraplast Plus (Sigma), and sectioned on a rotary microtome (Leica RM2235) at a thickness of 10 μm. The sections were mounted on silane-coated slides (sigma), deparaffinized in xylene, rehydrated in a graded ethanol-water series, and stained with toluidine blue. The intercalary meristem (2–3 mm above the node) and the elongation zone (3–10 mm above the node) collected from the elongating second internodes of main culms were plastic-embedded and sectioned for cytological observation as described^55^.

For DIC Observations, the widest part of leaf blades and the middle part of leaf sheaths were fixed in FAA (3.7% formaldehyde, 5% glacial acetic acid, 50% ethanol) followed by Clearing in 85% lactic acid for 48 hours. Leaf epidermal cells were observed with an Axio Imager A1 microscope (Zeiss) equipped with an AxioCam MRc5 digital camera (Zeiss) by using DIC optics. Root epidermal cells with root hair bulges were imaged by fluorescence microscopy (Axio Imager A1) with sample autofluorescence after fixation in FAA.

The stem elongation zone from the second internode of main culms were prepared and used for transmission electron microscopy of cell walls and scanning electron microscopy of cellulose microfibrils according to the described methods^56^. Angles of cellulose microfibrils were measured with the Image-Pro plus software as previously described^57^.

### Immunostaining

For immunofluorescence microscopy of tubulin and microtubule arrays in elongating cells, stem segments from the elongation zone of the second internode were hand-sectioned longitudinally with a razor blade and fixed immediately with 4% paraformaldehyde in PME (50 mM Pipes, 2 mM MgSO_4_, 2 mM EGTA). The fixed stem sections were hybridized with the mouse anti-β-tubulin antibody and then with the Alexa Fluor 488 goat anti-mouse IgG antibody using the described method^58^.

Immunostaining for OsKinesin-13A and tubulin were performed with root tips from 3-day-old seedlings, which were fixed with 4% (wt/vol) paraformaldehyde in PME buffer for 30 min at room temperature. After rinsing thoroughly with PME buffer, the root tips were incubated in PME containing 2% cellulase and 1% pectinase at 37°C for 30 min. The softened root tips were washed gently with PME, followed by squashing between slides and coverslips. The slides were frozen in liquid nitrogen and left to dry for 2 hours after the coverslips were flicked off. The squashed slides were covered with blocking buffer [3% BSA in PBST (135 mM NaCl, 25 mM KCl, 5 mM Na_2_HPO_4_, 2 mM KH_2_PO_4_, 0.05% Triton X-100)] and incubated in a humid chamber at room temperature for 1 hour. Afterwards, the slides were incubated overnight at 4°C with blocking buffer containing the 1/1000 diluted rabbit anti-OsKinesin-13 antibody and the 1/500 diluted mouse anti-β-tubulin antibody (Sigma), followed by 5 times washing with PBST. For colocalization analysis, the anti-β-tubulin antibody was replaced by a mouse anti-cis-Golgi 58K protein antibody (Sigma). The secondary hybridization was performed for 5 hours at room temperature with Alexa Fluor 594 goat anti-rabbit IgG (1/500 diluted in PBST) and Alexa Fluor 488 goat anti-mouse (or rat) IgG antibodies (Invitrogen). After washing 4 times with PBST and 1 time with PBS, the slides were counterstained with 1 μg/ml 4′-6-diamidino-2-phenylindole (DAPI, Sigma) and mounted in a VECTASHIELD Mounting Medium (Vector Laboratories). Immunostained cells were imaged using the Imager A1 microscope. The digital images were processed and merged with the Image-Pro plus software.

Immunogold electron microscopy (detailed in the Supplemental Methods) was performed using the described method^36^.

### Anti-microtubular drug treatment

Mature caryopses of WT and *sar1* were germinated on 1% agar under constant light at 30°C for 2 days. Germinated seedlings with roots shorter than 1 mm were transferred to 1% agar plates containing given concentrations of oryzalin (Sigma) or paclitaxel (Sigma). After 3 days of growth under constant light at 30°C, seedlings were transferred onto microscope slides and their roots were photographed with the Leica S8APO stereomicroscope (Leica) equipped with ProgRes C5 CCD (Analytik Jena AG). The digitized root images were analyzed with the ProgRes CapturePro 2.8.8 software for measuring root diameters.

To analyze changes in microtubule arrays after drug treatment, mature caryopses were germinated on water-saturated filter paper at 37°C for 1 day and then treated with 25 μM paclitaxel or 250 nM oryzalin at 37°C for 24 hours. Root tips from treated seedlings were fixed and immunostained for microscopy as described above.

### *In Vivo* Imaging of Microtubules

To obtain transgenic plants expressing GFP-tagged α-tubulin, a pUbi-GFP-α-tubulin plasmid was constructed by inserting the coding sequences of rice α-tubulin (LOC_Os03g51600.1) and EGFP into the *Bam*HI/*Spe*I and *Spe*I/*Bst*EII sites of the pUN1301 vector^59^, respectively. The rice α-tubulin fragment was RT-PCR amplified using primer pairs TubF (5′ GCCACTAGTATGAGGGAGTGCA TCTC 3′) and TubR (5′ATCGGTCACCCTAGTACTCGTCACCATC 3′). Transgenic WT and *sar1* plants containing GFP-α-tubulin were obtained by plant transformation^60^ using the *Agrobacterium tumefaciens* strain EHA105 harboring the constructed plasmid.

Root tips (about 1 cm) from one-month-old transgenic plants were incubated in PME at 23°C for at least 30 min and used to observe the behavior of individual microtubules *in vivo* with an Andor Revolution XD Spinning Disk Confocal System (Andor Technology). Cells with well-resolved individual microtubules (most of them were at the late elongation or differentiation stage) were used to analyze microtubule dynamics with the Image J software (http://imagej.nih.gov/ij/) according to the described method^61^.

### Tubulin Binding and Microtubule Depolymerization Assays

Sedimentation analysis of microtubule depolymerization was performed by mixing the purified GST (as a control), the OsKinesin-13A-motor protein, or the full-length OsKinesin-13A protein with 1 μM taxol-stabilized microtubules (Cytoskeleton Inc.) in a PEM-KOH buffer (80 mM PIPES, 1 mM EGTA, 1 mM MgCl_2_, 10 μM taxol, 30 mM KCl, pH 6.9) plus 2 mM ATP or AMPPNP. After 10 min incubation at 25°C, reaction mixtures were centrifuged at 25,000 g for 20 min to separate supernatants and pellets. Pellets generated from 100-μL reaction volumes were resuspended in 100 μL of ice-cold resuspension buffer (80 mM PIPES-KOH, 10 mM CaCl_2_, pH 6.9) by vigorous pipetting. Equivalent amounts of supernatants and pellets were analyzed by 12.5% SDS-PAGE, followed by Coomassie staining. The staining intensity was quantified with an Image-Pro Plus 5.1 software to determine the level of microtubules in supernatants and pellets.

For visual analysis of microtubule depolymerization, fluorescent microtubules were polymerized by incubating rhodamine-labeled tubulin (Cytoskeleton) with unlabeled tubulin (2:5) at 35°C for 20 min. The depolymerization assay was performed with 1 μM fluorescent microtubules as described. Reaction mixture was diluted in the PEM-KOH buffer containing an antifade reagent and transferred to poly-L-lysine coated slides (Sigma) for visualization under the Imager A1 microscope.

For tubulin binding assay, Sepharose beads bound to GST (as a control) or GST-OsKinesin-13A were incubated with 5 μM tubulin (Cytoskeleton) in a PEM-NaOH buffer (100 mM PIPES, 1 mM EGTA, 3 mM MgCl_2_, pH 6.9) containing 2 mM ATP (Merck) or AMPPNP (Sigma). After 1 h incubation at 4°C, beads were washed 3 times with the PEM-NaOH buffer and boiled in 1×SDS-PAGE sample buffer. The eluted proteins were separated on 12.5% SDS-PAGE gels, stained with Coomassie blue and photographed by a PowerLook 1120 scanner (Umax Technologies).

## Supplementary Material

Supplementary Information Supplemental Data

Supplementary Information Supplemental Movie 1

Supplementary Information Supplemental Movie 2

Supplementary Information Supplemental Movie 3

Supplementary Information Supplemental Movie 4

## Figures and Tables

**Table 1:** Size and weight of *sar1* grains, florets, and caryopses compared with WT

Sample			Length (mm) (%)	Width (mm) (%)	Thickness (mm) (%)	Weight (g) (%)^d^
Mature grains^a^		WT	7.39 ± 0.20 (100)	3.40 ± 0.13 (100)	2.33 ± 0.07 (100)	27.61 ± 0.91 (100)
		*sar1*	4.91 ± 0.19 (66)	3.82 ± 0.19 (113)	2.46 ± 0.08 (106)	19.26 ± 0.20 (70)
		*P* value	3.1E-163	8.3E-44	3.2E-27	1.5E-06
Mature florets^b^		WT	7.69 ± 0.27 (100)	3.41 ± 0.15 (100)	1.67 ± 0.13 (100)	-
		*sar1*	5.55 ± 0.24 (72)	3.64 ± 0.24 (107)	1.76 ± 0.14 (105)	-
		*P* value	7.7E-130	1.0E-13	1.1E-05	-
Mature caryopses^a^		WT	5.13 ± 0.12 (100)	2.96 ± 0.09 (100)	2.08 ± 0.08 (100)	22.78 ± 0.90 (100)
		*sar1*	3.21 ± 0.14 (63)	3.16 ± 0.15 (107)	2.22 ± 0.10 (107)	15.32 ± 0.16 (67)
		*P* value	2.7E-172	4.8E-24	1.1E-22	3.9E-03
The glume-cutting experiment	Remaining glumes^c^	WT	4.33 ± 0.38 (100)	3.05 ± 0.18 (100)	2.21 ± 0.20 (100)	-
		*sar1*	4.33 ± 0.31 (100)	3.66 ± 0.16 (120)	2.57 ± 0.21 (117)	-
		*P* value	9.9E-01	1.8E-26	1.7E-11	-
	Caryopses^c^	WT	4.90 ± 0.21 (100)	1.84 ± 0.15 (100)	1.53 ± 0.15 (100)	-
		*sar1*	4.98 ± 0.31 (102)	2.18 ± 0.23 (118)	1.84 ± 0.23 (120)	-
		*P* value	1.5E-01	1.2E-10	5.1E-10	-

Means ± standard deviations (SD) are shown. The numbers in parentheses indicate the percentage ratio of *sar1* to WT. The symbol ‘-’ indicates data were not determined. A Student's *t*-test was used to generate the *P* values.

^a^length, width and thickness were determined from 100-103 completely filled grains or caryopses.

^b^length, width and thickness were determined from 102-103 mature florets.

^c^Sample number of the glume-cutting experiment is 40.

^d^The weight of 100 completely filled grains or caryopses was calculated and converted to 1000-seed weight; data shown in the table represent the mean of three independent measurements.

**Table 2:** Cell size of *sar1* internodes, leaves, and florets compared with WT

Sample		Cell length (μm) (%)	Cell width (μm) (%)	Cell area (μm^2^) (%)
Parenchymal cells in the elongation zone of the second internode	WT	80.4 ± 29 (100)	33.7 ± 10 (100)	-
	*sar1*	57.3 ± 20 (71)	33.8 ± 9 (100)	-
	*P* value	2.2E-31	8.6E-01	-
Epidermal cells in the sheath of the flag leaf	WT	90.2 ± 23.7 (100)	15.7 ± 2.1 (100)	-
	*sar1*	65.1 ± 18.2 (72)	15.2 ± 1.7 (97)	-
	*P* value	4.7E-18	2.3E-01	-
Epidermal cells in the blade of the flag leaf	WT	78.3 ± 21.3 (100)	16.3 ± 3.5 (100)	-
	*sar1*	58.4 ± 17.6 (75)	17.5 ± 2.9 (107)	-
	*P* value	2.8E-12	2.1E-01	-
Sclerenchyma cells in the lemma of mature florets	WT	294.6 ± 98.6 (100)	-	98.9 ± 44.7 (100)
	*sar1*	213.9 ± 79.0 (73)	-	201.4 ± 118.0 (204)
	*P* value	2.8E-06	-	2.4E-29
Root epidermal cells with visible root hair bulges	WT	102.5 ± 21.0 (100)	-	-
	*sar1*	71.3 ± 18.9 (70)	-	-
	*P* value	5.6E-143	-	-

Means ± SD are shown. The numbers in parentheses indicate the percentage ratio of *sar1* to WT. The symbol ‘-’ indicates data were not determined. The Student's *t*-test was used to generate the *P* values. Sample number = 50-937 cells from 3-10 independent tissues.

**Table 3:** Parameters of microtubule dynamic instability in *sar1* root cells compared with WT

Sample	Rate (μm/min) (%)		Transition frequencies (events/min) (%)		Percent time in phase (%) (%)			Dynamicity (μm/min) (%)
	Growth	Shrinkage	Rescue	Catastrophe	Growth	Shrinkage	Pause	
WT	3.76 ± 0.98 (100)	14.18 ± 4.01 (100)	3.33 (100)	0.78 (100)	66.02 (100)	15.62 (100)	18.36 (100)	5.02 (100)
*sar1*	2.82 ± 0.53 (75)	16.64 ± 5.35 (117)	3.39 (102)	0.60 (77)	66.09 (100)	13.08 (84)	20.83 (113)	4.69 (93)
*P* value	3.1E-16	1.2E-04	-	-	-	-	-	-

Means ± SD are shown. The numbers in parentheses indicate the percentage ratio of *sar1* to WT. The *P* values were generated by the Student's *t*-test. The symbol ‘-’ indicates values were not determined. Sample number = 174 microtubules in 55 cells from 4 transgenic WT lines and 187 microtubules in 70 cells from 8 transgenic *sar1* lines. Dynamicity is calculated as the sum of the total length grown and shortened divided by the total time of observation for all microtubules.

**Table 4:** Localization of OsKinesin-13A in cells

	Anti-OsKinesin-13A Antibody	Preimmune Serum
**Average Number of Gold Particles**		
Particles/cell	58.17 ± 3.08	6.25 ± 0.59
Particles/vesicle	1.60 ± 0.02	-
**Labeling Distribution (in Percent) in Cells**		
Vesicles	52.51 ± 1.65	1.08 ± 0.75
Cytoplasm	36.57 ± 1.74	82.84 ± 5.80
Cell membrane	5.46 ± 0.49	0.50 ± 0.50
Golgi stacks	2.29 ± 0.39	-
Endoplasmic reticulum	1.74 ± 0.53	-
Other organelles	1.42 ± 0.42^a^	15.58 ± 5.85^b^
**Distribution (in Percent) of Labeled Vesicles in Cells**		
In the vicinity of cell membrane^c^	37.96 ± 2.71	-
In the vicinity of Golgi stacks^c^	32.04 ± 2.12	-
Other areas in the cytoplasm	30.00 ± 2.50	-

Means ± standard errors of the mean are shown. Sample numbers for immunogold labeling with the anti-OsKinesin-13a antibody and the preimmune serum (negative control) were 41 and 20 root tips cells of WT seedlings, respectively. The symbol ‘-’ indicates signals were not detected.

^a^Organelles included vacuoles, mitochondria and nuclei.

^b^Organelles were nuclei.

^c^Labeled vesicles located in the area around and near (straight-line distance <1 μm) cell membrane or Golgi stacks.
