# Supplementary material for: OsKinesin-13A Is an Active Microtubule Depolymerase Involved in Glume Length Regulation via Affecting Cell Elongation
Source: Sci Rep. 2015 Mar 25;5:9457. doi: 10.1038/srep09457 (PMC4373686; doi:10.1038/srep09457)
Supplement: Supplementary Information — Supplemental Data [file srep09457-s1.docx]

**OsKinesin-13A Is an Active Microtubule Depolymerase Involved in Glume Length Regulation via Affecting Cell Elongation**

Zhu Yun Deng, Ling Tong Liu, Tang Li, Song Yan, Bai Jian Kuang, Shan Jin Huang, Chang Jie Yan, Tai Wang

**Supplemental Figures**

**
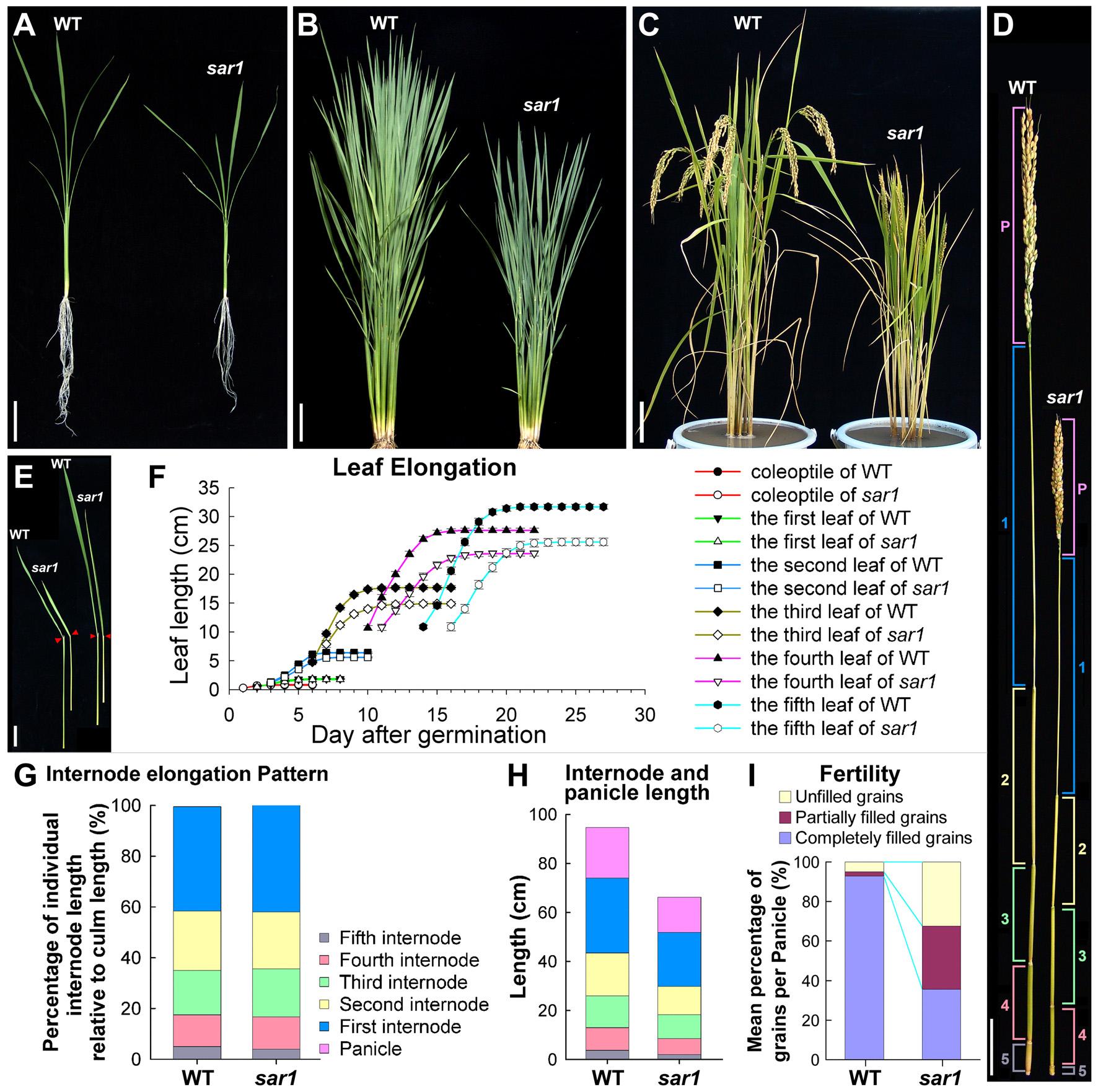
**

**Supplemental Figure 1. The *sar1* mutant is semi-dwarfed and partial-sterile**

**(A-C) the *sar1* plants showed reduced height during the juvenile vegetative phase** (A, twenty-eight-day-old seedlings, bar=5 cm; leaves and roots of the *sar1* seedling were shorter than those of WT)**, the adult vegetative phase** (B, bar=10 cm)**, and the reproductive phase** (C, bar=10 cm)**.**

**(D-H) Shortened leaves and internodes resulted in semi-dwarfism in *sar1*.** The increase in plant height at the vegetative stage is dependent on the elongation of leaves. We observed the elongation process of the first five leaves and found that for the coleoptiles and the first leaves, growth curves of *sar1* and WT almost completely overlapped with each other (F, error bars indicate standard errors of the mean). But the second to fifth leaves of *sar1* were shorter than those of WT at every growth stage despite having growth patterns similar to WT (F). These observations suggest the whole process of elongation is defective in *sar1* leaves except for the coleoptile and the first leaf. Plant height at the mature stage is determined by the length of fully elongated stems and mature leaves. We measured the final length of the upper five internodes on the main culms and demonstrated that although the upper five internodes of *sar1* were all elongated during the reproductive stage (D, the letter ‘P’ denotes the panicle and digits denote the number of each internode; bar=5cm) and the elongation pattern was similar to WT (G), their length was proportionally diminished compared with WT (D and H). Similarly, panicles (D and H) and leaf blades and sheaths of *sar1* at the mature stage were shortened (Supplemental Table 1; E shows the flag and penultimate leaves; red arrowheads indicate the lamina joints, which connect the blades and the sheaths of leaves; bar=5 cm).

**(I) the fertility of *sar1* was significantly decreased.**

**
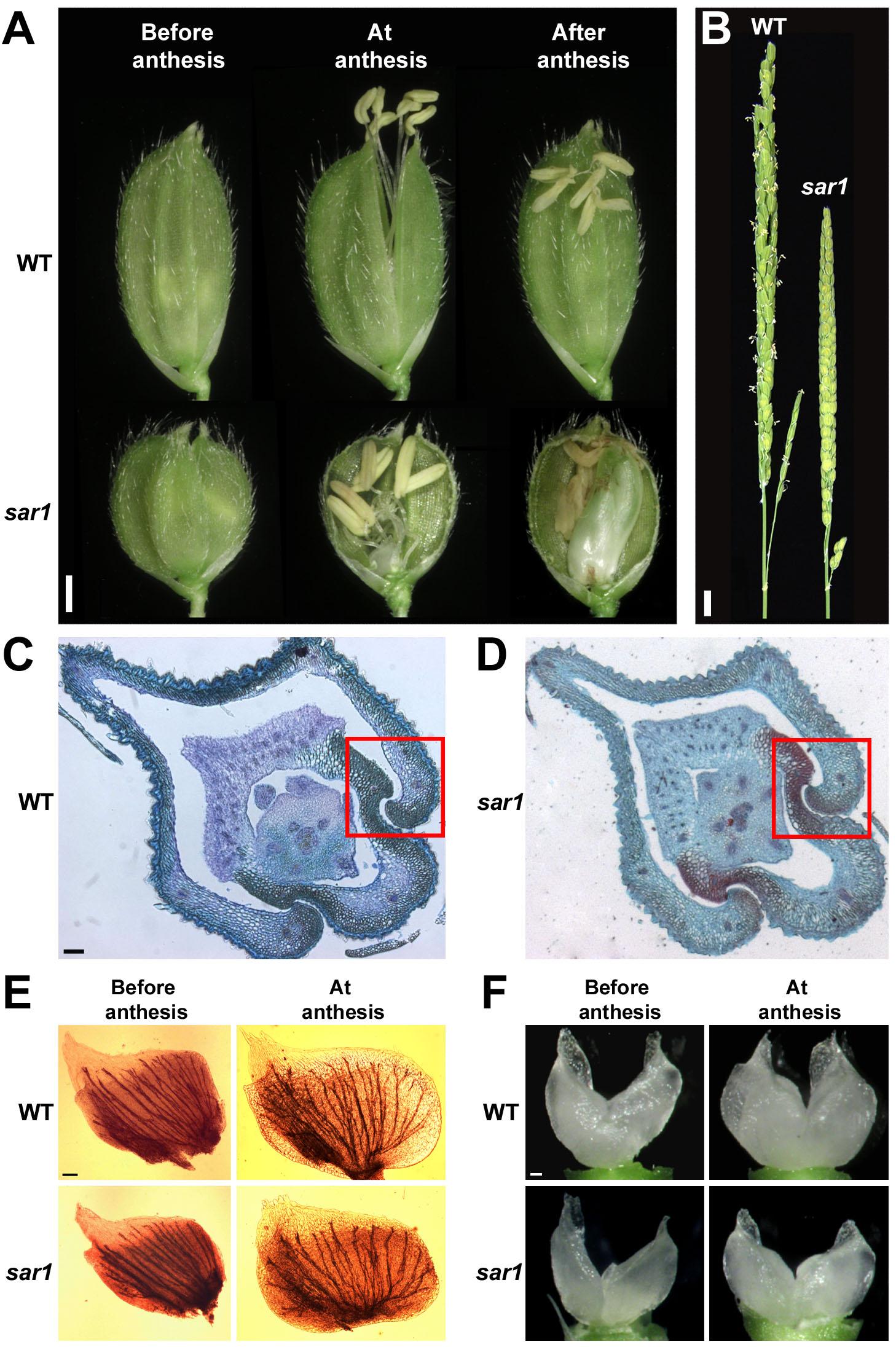
**

**Supplemental Figure 2. The *sar1* mutant is cleistogamous**.

**(A and B) the *sar1* plants finished pollination without opening their florets.** When a WT floret flowers, the lemma and the palea (the glumes) are thrust apart by the swelling of the two lodicules and the six anthers protrude from the glumes by the elongation of their filaments (A, bar=1 mm). Therefore, most of the florets on WT panicles had open glumes and their anthers were visible between or above the glumes at the time of flowering but were left outside the glumes after flowering (A and B). In contrast, open glumes were not observed on *sar1* panicles at the flowering stage and their anthers were completely enclosed in the glumes (B, bar=1 cm), even though the filaments of anthers elongated normally (A). These enclosed anthers were still visible inside the glumes during caryopsis development (A).

**(C-F**) **cleistogamy is mainly caused by size reduction in lodicules.** Floret opening in rice is a process that its lemma is forced to separate from its palea by its two swelling lodicules. Morphological abnormalities in florets and/or in the fusion between lemmas and paleas could lead to cleistogamy ([Maeng et al., 2006](#_heading=h.2jxsxqh); [Yoshida et al., 2007](#_heading=h.2xcytpi)). In *sar1*, floral organ identity was not changed (Supplemental Figure 2A) and the connections between lemmas and paleas were normal (shown in red squares in C and D, bar=100 µm). Therefore, these two factors could not obstruct floret opening. In addition, floret opening depends mainly on the morphology and size of the lodicules ([Li et al., 2005](#_heading=h.44sinio); [Maeng et al., 2006](#_heading=h.2jxsxqh); [Yoshida et al., 2007](#_heading=h.2xcytpi)). The lodicules of *sar1* showed normal morphology (F, bar=100 µm) and contained a similar number of vascular bundles as WT (E, bar=100 µm), enabling them to swell. However, their size was reduced both before and at anthesis compared to WT (E and F; Supplemental Table 3), suggesting the reduction in lodicule size may relate to the cleistogamous phenotype in *sar1*.

**
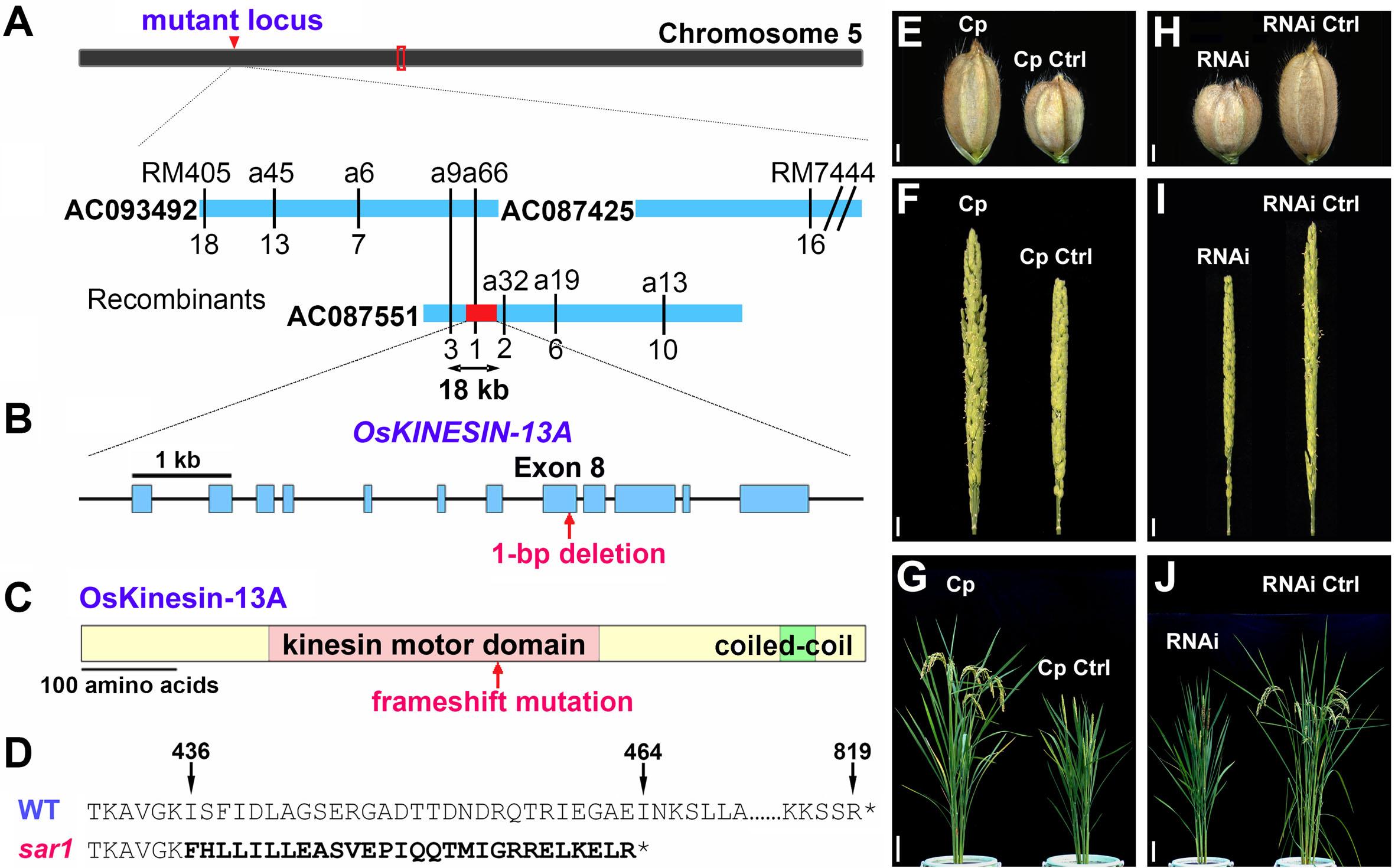
**

**Supplemental Figure 3. A deletion mutation in *OsKINESIN-13A* leads to *sar1* phenotypes**

**(A-D) Positional cloning was performed to identify the mutant gene of *sar1*.** From the F_2_ population generated by crossing *sar1* with Nanjing 11, a total of 745 mutant plants were obtained and used for genetic analysis. The mutant locus was mapped between the molecular markers RM405 and RM7444 on chromosome 5, with the distance of 0.42 cM and 0.84 cM, respectively (A). Further mapping using adjacent Sequence-tagged-site (STS) markers (Supplemental Table 5) indicated that the mutant locus was in BAC clone AC087551 and its position was gradually narrowed to an 18 kb DNA region (A). According to the Rice Genome Annotation Project Database (RGAP, http://rice.plantbiology.msu.edu/), this DNA region comprises only one gene (B) (RGAP ID: LOC_Os05g06280 or Rice Annotation Project ID: [Os05g0154700](http://rapdb.dna.affrc.go.jp/viewer/gbrowse_details/build5?name=Os05g0154700)), which encodes the rice kinesin-13 family member, OsKinesin-13A (C) ([Guo et al., 2009](#_heading=h.2s8eyo1)). The OsKinesin-13A protein consists of 819 amino acid residues and contains a kinesin motor domain (amino acid 197-541, the red box in C) in the middle and a coiled-coil domain (731-767, the green box in C) in the carboxyl terminus (C) ([Kitagawa et al., 2010](#_heading=h.lnxbz9)). In the *sar1* mutant, a deletion of a single adenine nucleotide (the red arrow in B) was found in the eighth exon (the blue boxes in B) of the *OsKinesin-13A* gene (B). This point deletion caused a reading frame shift (its beginning is indicated by the red arrow in C) from codon 436 and created a stop codon at codon 464, resulting in a truncated protein with presumably disrupted kinesin motor domain (C). (D) The amino acid sequence of the truncated OsKinesin-13A protein in *sar1* compared with the normal protein in WT. The carboxyl-terminal 384 amino acids of the normal protein are replaced by 28 novel amino acids.

**(E-J) Both complementation and RNAi knockdown analyses confirm that the abnormal phenotypes of *sar1* were caused by the mutation in the *OsKINESIN-13A* gene.** For complementation analysis of the *sar1* mutants, the full-length *OsKINESIN-13A* ORF driven by the maize Ubi-1 promoter was introduced into the *sar1* mutants to generate complementation lines using the *Agrobacterium tumefaciens*-mediated transformation method. Among the 57 independent complementation lines (T0 generation), 65% of them recovered to WT grain shape, 28% of them had grains longer than *sar1* but shorter than WT. In contrast, all of the 22 independent complementation control lines (T0 generation) remained their *sar1* grain shapes. Because transgenic T0 plants are directly derived from rice calli, they themselves have abnormalities in plant height, grain production and other phenotypes. Therefore, further phenotypic analysis was performed using T1 rice plants grown from seeds of T0 plants. One complementation line with WT-shaped grains (Cp L18) and one complementation control line (Cp Ctrl L5) were randomly selected for statistic analysis of the four phenotypes at T1 generation. Compared to the T1 control plants of Cp Ctrl L5, T1 plants of Cp L18 showed the wild-type phenotypes (E-G, Supplemental Table 4). Mature grains of Cp displayed WT shape while mature grains of Cp Ctrl were still shorter in length and rounder in shape, similar to the *sar1* grains shown in Figure 1A (E, scale bar=1 mm). Yellow anthers were visible on Cp panicles at the flowering stage but invisible on Cp Ctrl panicles (F, scale bar=1 cm). Cp plants at the mature stage were higher than Cp Ctrl plants and their panicles bent down compared with the upright panicles of Cp Ctrls (G, scale bar=10 cm). These observations indicate the multifaceted defects of the *sar1* mutant could be rescued by expression of the full-length OsKinesin-13A protein. To specifically knockdown the expression of *OsKINESIN-13A* in WT plants, we established double-stranded RNA-mediated interference (RNAi) transgenic lines by introducing the *OsKINESIN-13A*-unique sense and antisense cDNA fragments, spaced by a rice intron, into the WT genome. The RNAi plants (T1 generation) had phenotypes similar to the *sar1* mutants (H-J, Supplemental Table 4). Mature grains of RNAi showed *sar1* morphology, which were shorter in length and rounder in shape; while mature grains of RNAi Ctrl were similar to WT (H, scale bar=1 mm). Yellow anthers were invisible on shortened RNAi panicles at the flowering stage but visible on RNAi Ctrl panicles (I, scale bar=1 cm). RNAi plants at the mature stage were shorter than RNAi Ctrl plants and their panicles were upright compared with the bent panicles of RNAi Ctrls (J, scale bar=10 cm). These results imply knockdown of the *OsKINESIN-13A* gene in WT plants induced *sar1* phenotypes.

**
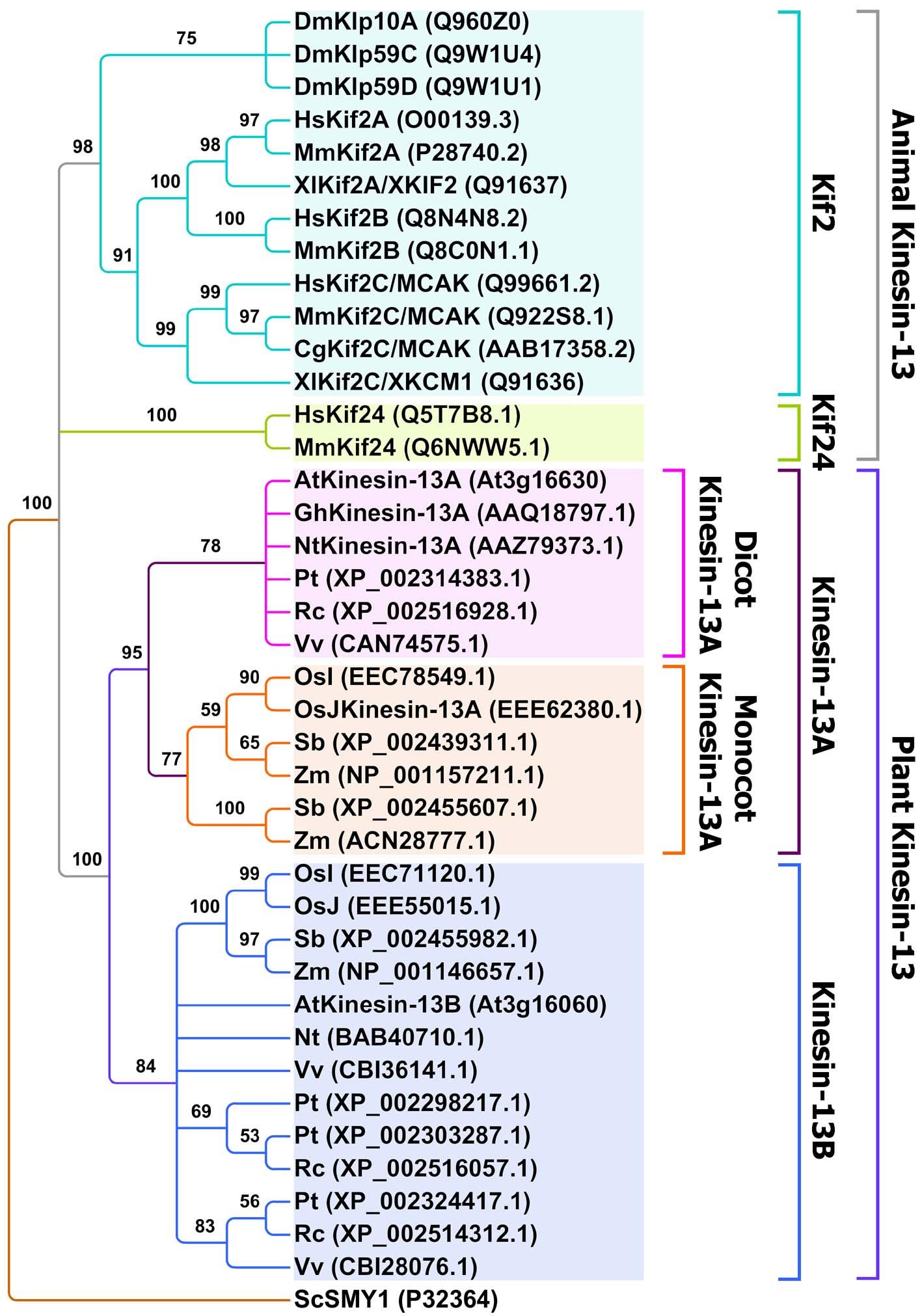
**

**Supplemental Figure 4. Phylogenetic tree of plant Kinesin-13 proteins.**

Known proteins are denoted by “protein name (NCBI or UniProtKB accession number)”; unknown proteins are denoted by “species abbreviation (accession number)”. The numbers at nodes represent bootstrap probability for each node out of 1000 bootstrap resamplings. Species abbreviations: At, *Arabidopsis thaliana*; Cg, *Cricetulus griseus*; Dm, *Drosophila melanogaster*; Gh, *Gossypium hirsutum*; Hs, *Homo sapiens*; Mm, *Mus musculus*; Nt, *Nicotiana tabacum*; OsI, *Oryza sativa ssp. Indica*; OsJ, *Oryza sativa ssp*. *Japonica*; Pt, *Populus trichocarpa*; Rc, *Ricinus communis*; Sc, *Saccharomyces cerevisiae*; Sb, *Sorghum bicolor*; Vv, *Vitis vinifera*; Xl, *Xenopus laevis*; Zm, *Zea mays*.


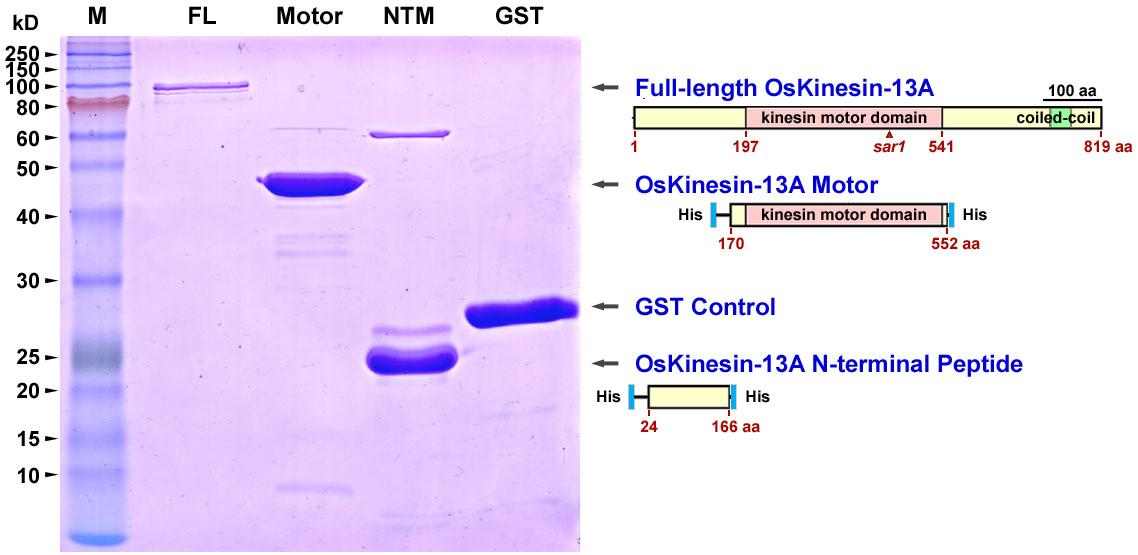


**Supplemental Figure 5. Purified recombinant proteins for biochemical studies**

In the 12.5% SDS-PAGE gel, lanes FL, Motor, NTM and GST were loaded with the purified samples of the full-length OsKinesin-13A protein (1μg), the His-tagged OsKinesin-13A motor protein (8 μg), the His-tagged N-terminal peptide (7 μg) and the glutathione S-transferase (GST) control protein (8 μg), respectively. Schematic diagrams on the right side of the gel show their protein domains. The pink and green boxes show the kinesin domain and the coiled-coil domain, respectively; other amino acid sequences of OsKinesin-13A are shown by yellow boxes. Thin black lines show amino acid residues (aa) expressed from expression vectors. The blue blocks show hexahistidine tags (His). Red numbers indicates the residue position on the OsKinesin-13A protein. Molecular-weight markers are in Lane M and their kilodalton (kD) values are indicated on the left side. Red arrowhead and word “*sar1*” indicates the mutation site of the *sar1* mutant.


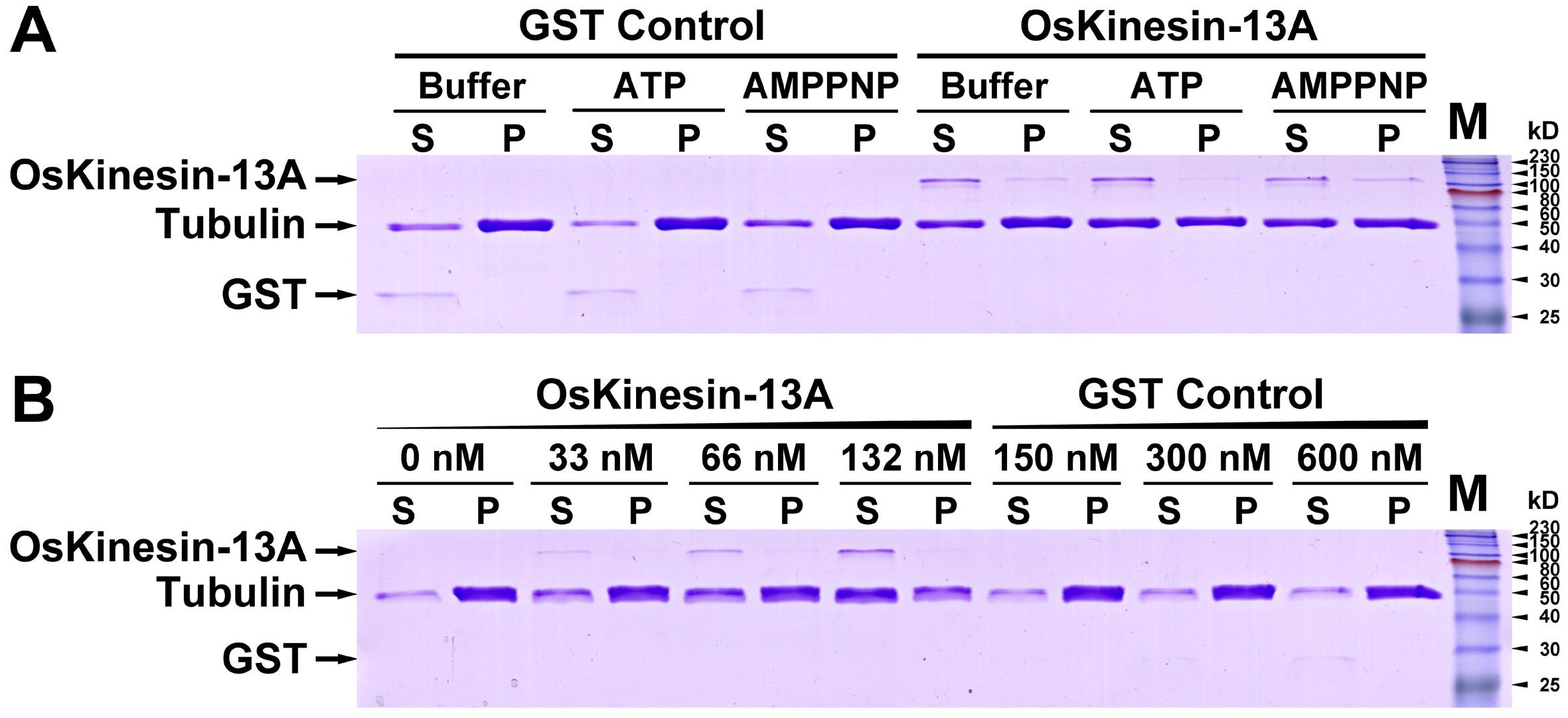


**Supplemental Figure 6. Full-length OsKinesin-13A protein depolymerizes microtubules *in vitro***

(A) Full-length OsKinesin-13A protein depolymerizes microtubules in the presence of ATP or AMPPNP. Microtubule depolymerization reactions were performed as described in the legend of Figure 7C, except 132 nM full-length OsKinesin-13A protein (OsKinesin-13A) was used instead of OsKinesin-13A-motor (1.6 μM). The bands of the full-length OsKinesin-13A protein are visible in gels. (B) The microtubule-depolymerizing activity of the full-length OsKinesin-13A protein is concentration dependent. Microtubule depolymerization reactions were performed using indicated amounts of GST (150, 300 and 600 nM) or full-length OsKinesin-13A protein (0, 33, 66 and 132 nM) as described in legends of Figure 7D. Compared to OsKinesin-13A-motor, the full-length OsKinesin-13A protein showed a higher level of microtubule depolymerization activity. For example, 400 nM OsKinesin-13A-motor resulted in the mean percentage of tubulin in the supernatant versus supernatant and pellet [tubulin S/(S+P) %, triple replicates] increasing from 22.0±3.0% (in the presence of GST control) to 59.5±0.9%. A similar increase in tubulin release [tubulin S/(S+P) % increased from 12.8±3.1% (GST control) to 56.9±3.1%, triple replicates) can be induced by only 132 nM full-length OsKinesin-13A proteins.


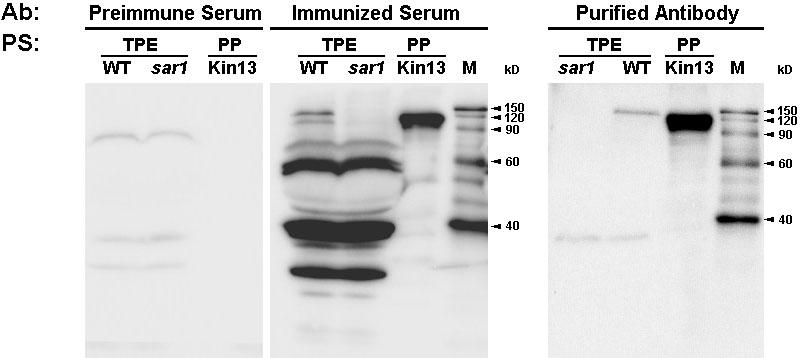


**Supplemental Figure 7. Western hybridization validates the specificity of the anti-OsKinesin-13A antibody**

For the immunofluorescence and immunogold methods, the prokaryotic-expressed and affinity-purified N-terminal peptide (142 amino acids) of OsKinesin-13A (Supplemental Figure 5) was used as immunogen to produce the rabbit anti-OsKinesin-13A polyclonal serum (immunized serum). Western hybridization showed in total protein extracts (TPE) isolated from wild-type rice seedlings, the produced serum recognized two protein bands between 90 and 120 kDa, corresponding to the size range of the purified full-length OsKinesin-13A protein (Kin13) used in the depolymerization assay. In contrast, the two protein bands were absent when TPE from WT seedlings were replaced by those from *sar1* seedlings, or when the Western blot was probed with the preimmune rabbit serum (preimmune serum), indicating one of the two bands may be the OsKinesin-13A protein. Therefore, we affinity purified this polyclonal antiserum with the immunogen peptide, followed by further verification of antibody specificity using Western hybridization. The purified antibody specifically detected the purified full-length OsKinesin-13A protein (Kin13). The corresponding protein band was present in TPE of WT but absent in TPE of *sar1*, validating the specificity of the purified antibody. An additional protein band with a molecular weight below 40 kD was detected in both WT and *sar1* protein extracts. This low-molecular-weight protein might be a relatively stable degraded product of the full-length (in WT) and truncated (in *sar1*) OsKinesin-13A proteins. Ab, antibody; PS, protein sample; PP, purified protein; M, protein molecular weight markers for Western blotting (EasySee Western Marker, Transgen Biotech); kD, kiloDalton.

**
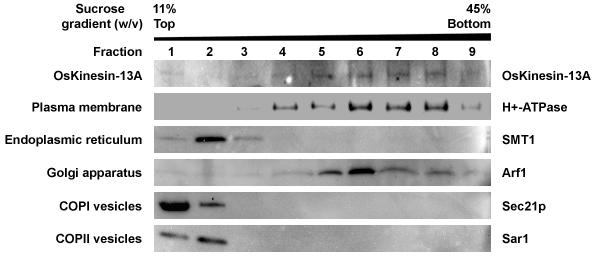
**

**Supplemental Figure 8. Distribution of OsKinesin-13A in microsomal fractions**

Microsomes were prepared from rice seedlings and further separated by discontinuous sucrose density gradient ultracentrifugation. Centrifuged gradients were fractionated from top to bottom into 9 equal fractions. The fractions were probed by western blotting with the antibodies against marker proteins for the plasma membrane (H^+^-ATPase), endoplasmic reticulum (Sterol methyltransferase1, SMT1), Golgi apparatus (ADP-ribosylation factor1, Arf1), COPI (coat protein I)-coated vesicles (Sec21p), and COPII-coated vesicles (Secretion-associated and Ras-related protein 1, Sar1), as well as with the anti-OsKinesin-13A antibody.

**
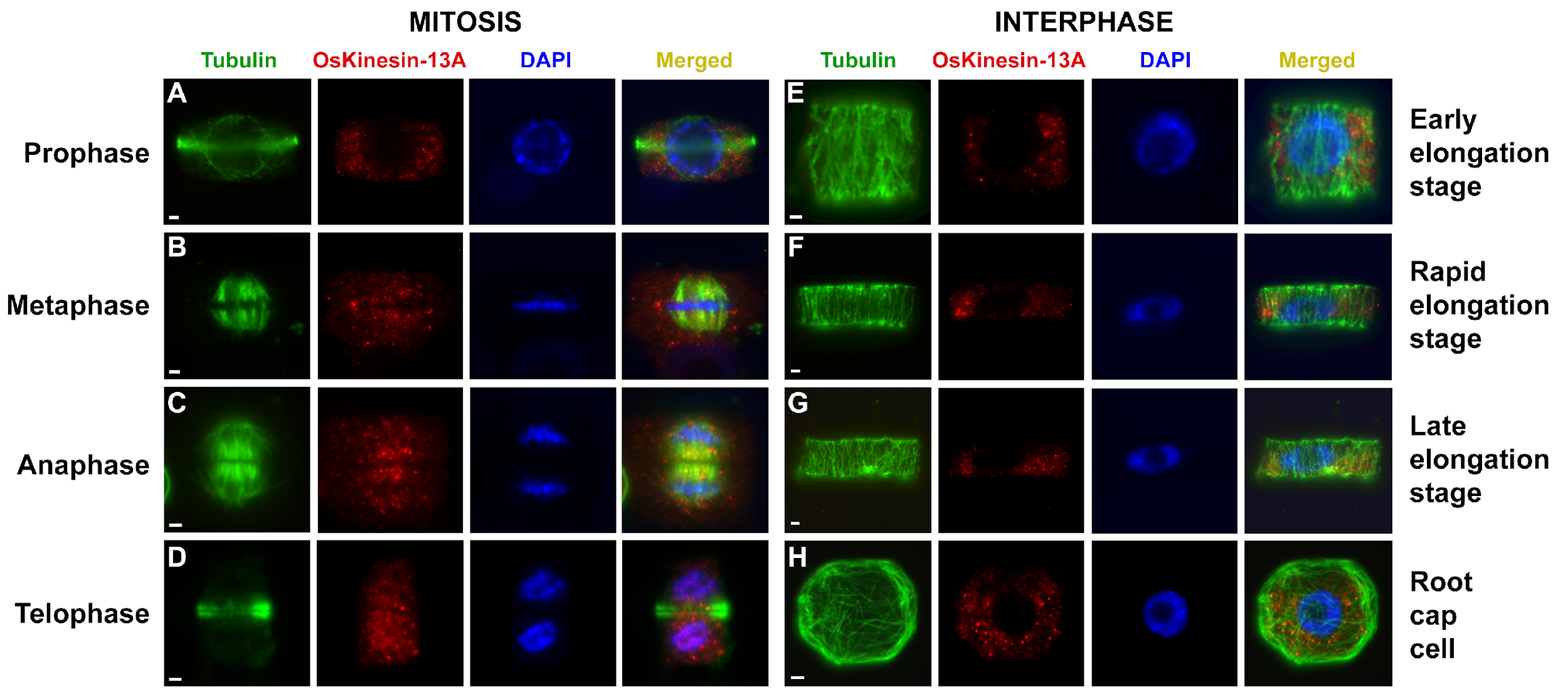
**

**Supplemental Figure 9. Subcellular localization of OsKinesin-13A in different types of cells**

OsKinesin-13A displayed a punctate distribution throughout the cytoplasm in both mitotic (A-D) and interphase (E-H) cells. WT root tip cells were hybridized with antibodies against tubulin (green) and OsKinesin-13A (red). The nuclei were counterstained with DAPI (blue). Bar =1 μm in (A) to (H).

**Supplemental Tables**

**Supplemental Table 1.** Length and width of *sar1* leaf blades and sheaths compared with WT

| **Sample** | | **Measured items** | **Length or width of WT** (cm) (%) | **Sample number of WT** | **Length or width of *sar1*** (cm) (%) | **Sample number of *sar1*** | ***P* value** |
| --- | --- | --- | --- | --- | --- | --- | --- |
| Leaves at the juvenile vegetative stage | The second leaf | Blade length | 1.9 ± 0.2 (100) | 36 | 1.4 ± 0.2 (73) | 37 | 1.7E-14 |
|  |  | Sheath length | 4.2 ± 0.5 (100) | 36 | 4.0 ± 0.5 (94) | 37 | 6.1E-02 |
|  | The third leaf | Blade length | 8.6 ±1.0 (100) | 39 | 7.1 ± 1.4 (83) | 40 | 1.2E-06 |
|  |  | Sheath length | 8.7 ± 1.0 (100) | 39 | 8.1 ± 1.0 (92) | 40 | 4.5E-03 |
|  | The fourth leaf | Blade length | 18.0 ±1.5 (100) | 36 | 16.9 ± 2.0 (94) | 36 | 1.0E-02 |
|  |  | Sheath length | 10.5 ± 0.9 (100) | 36 | 8.4 ± 1.2 (80) | 36 | 8.4E-12 |
|  | The fifth leaf | Blade length | 20.6 ± 2.7 (100) | 36 | 17.1 ± 2.9 (83) | 36 | 9.2E-07 |
|  |  | Sheath length | 9.9 ± 0.7 (100) | 36 | 8.2 ± 0.7 (83) | 36 | 1.2E-14 |
| The upper five leaves at the mature stage | The flag leaf | Blade length | 34.7 ± 8.0 (100) | 22 | 20.8 ± 5.1 (60) | 40 | 3.2E-08 |
|  |  | Blade width | 1.5 ± 0.1 (100) | 9 | 1.5 ± 0.1 (101) | 9 | 5.5E-01 |
|  |  | Sheath length | 30.9 ± 4.8 (100) | 22 | 21.2 ± 2.4 (69) | 40 | 1.6E-09 |
|  | The penultimate leaf | Blade length | 49.0 ± 6.9 (100) | 22 | 36.1 ± 6.7 (74) | 40 | 8.5E-09 |
|  |  | Blade width | 1.3 ± 0.1 (100) | 9 | 1.3 ± 0.2 (103) | 9 | 5.5E-01 |
|  |  | Sheath length | 26.6 ± 3.0 (100) | 22 | 19.5 ± 2.3 (73) | 40 | 2.5E-11 |
|  | The third leaf from the top of plant | Blade length | 55.1 ± 5.8 (100) | 40 | 44.7 ± 5.3 (81) | 56 | 8.9E-14 |
|  |  | Blade width | 1.2 ± 0.1 (100) | 27 | 1.2 ± 0.1 (95) | 25 | 6.7E-02 |
|  |  | Sheath length | 27.6 ± 2.1 (100) | 40 | 22.3 ± 2.6 (81) | 56 | 9.8E-19 |
|  | The fourth leaf from the top of plant | Blade length | 59.5 ± 4.5 (100) | 27 | 45.0 ± 8.7 (76) | 26 | 4.6E-09 |
|  |  | Blade width | 1.2 ± 0.1 (100) | 27 | 1.1 ± 0.2 (96) | 26 | 2.4E-01 |
|  |  | Sheath length | 28.7 ± 1.4 (100) | 27 | 24.4 ± 2.2 (85) | 26 | 9.3E-11 |
|  | The fifth leaf from the top of plant | Blade length | 57.3 ± 4.1 (100) | 21 | 43.9 ± 9.3 (77) | 20 | 3.3E-06 |
|  |  | Blade width | 1.2 ± 0.1 (100) | 21 | 1.1 ± 0.2 (94) | 20 | 1.5E-01 |
|  |  | Sheath length | 29.0 ± 1.8 (100) | 21 | 23.5 ± 3.1 (81) | 20 | 9.6E-08 |

Means ± standard deviations (SD) are shown. The numbers in parentheses indicate the percentage ratio of *sar1* to WT. A Student’s *t*-test was used to generate the *P* values.

| **Sample** | **Length of the**  **first embryonic crown root** (cm) (%) | **Length of the**  **second embryonic crown root** (cm) (%) | **Average length of all crown roots** (cm) (%) | | |
| --- | --- | --- | --- | --- | --- |
|  | **Seven days**  **after germination** | **Seven days**  **after Germination** | **Fourteen days after germination** | **Twenty-one days after germination** | **Twenty-eight days after germination** |
| WT | 5.2 ± 0.7 (100) | 4.9 ± 0.9 (100) | 8.5 ± 2.5 (100) | 11.1 ± 3.0 (100) | 11.8 ± 5.0 (100) |
| *sar1* | 4.3 ± 1.2 (83) | 4.1 ± 1.4 (85) | 5.4 ± 2.5 (63) | 9.2 ± 2.6 (83) | 9.2 ± 4.7 (78) |
| *P* value | 6.1E-03 | 6.0E-02 | 1.7E-05 | 1.9E-04 | 4.2E-05 |

**Supplemental Table 2.** Length of *sar1* roots compared with WT

Means ± SD are shown. The numbers in parentheses indicate the percentage ratio of sar1 to WT. A Student’s t-test was used to generate the P values. Sample number=18-136 roots from 5-23 independent seedlings.

| **Sample** | **Before anthesis** | | |  | **At anthesis** | | |
| --- | --- | --- | --- | --- | --- | --- | --- |
|  | **Lodicule length** (mm) (%) | **Lodicule width** (mm) (%) | **Length/width ratio of lodicules** (%) |  | **Lodicule length**  (mm) (%) | **Lodicule width**  (mm) (%) | **Length/width ratio of lodicules** (%) |
| WT | 1.19 ± 0.08 (100) | 0.70 ± 0.06 (100) | 1.71 ± 0.16 (100) |  | 1.20 ± 0.07 (100) | 1.04 ± 0.08 (100) | 1.16 ± 0.09 (100) |
| *sar1* | 0.96 ± 0.07 (81) | 0.69 ± 0.07 (99) | 1.40 ± 0.13 (82) |  | 1.06 ± 0.07 (88) | 0.95 ± 0.09 (91) | 1.12 ± 0.12 (97) |
| *P* value | 1.4E-27 | 4.5E-01 | 2.4E-18 |  | 1.1E-17 | 1.1E-06 | 3.8E-02 |

**Supplemental Table 3.** Size of *sar1* lodicules compared with WT

Means ± SE are shown. The numbers in parentheses indicate the percentage ratio of *sar1* to WT. A Student’s *t*-test was used to generate the *P* values. Sample number=50-65.

**Supplemental Table 4.** Phenotypes of the complementation line and its control, and the RNAi line and its control compared with those of WT and *sar1*

| **Phenotypes** | | **WT** | ***sar1*** | **Cp** | **Cp Ctrl** | **RNAi** | **RNAi Ctrl** |
| --- | --- | --- | --- | --- | --- | --- | --- |
| **Grain size** | **Length (mm) (%)** | 7.39 ± 0.02 (100) | 4.91 ± 0.02 (66) | 7.49 ± 0.08 (101) | 4.92 ± 0.10 (67) | 5.01 ± 0.05 (68) | 7.38 ± 0.08 (100) |
|  | **Width (mm) (%)** | 3.40 ± 0.01 (100) | 3.82 ± 0.02 (113) | 3.20 ± 0.03 (94) | 3.63 ± 0.08 (107) | 3.74 ± 0.03  (110) | 3.32 ± 0.04  (98) |
|  | **Thickness (mm) (%)** | 2.33 ± 0.01 (100) | 2.46 ± 0.01 (106) | 2.16 ± 0.02 (93) | 2.35 ± 0.05 (101) | 2.46 ± 0.02 (106) | 2.27 ± 0.03 (97) |
| **Plant height (cm) (%)** | | 127.0 ± 3.5 (100) | 104.9 ± 1.3 (83) | 126.1 ± 0.7 (99) | 102.8 ± 2.6 (81) | 103.8 ± 3.1 (82) | 125.9 ± 2.8 (99) |
| **Grain production** | **Fully filled grains (%) (%)** | 92.9 ± 1.0 (100) | 35.6 ± 2.6 (38) | 82.7 ± 2.2 (89) | 39.0 ± 1.8 (42) | 28.4 ± 2.7 (31) | 82.7 ± 1.9 (89) |
|  | **Partially filled grains (%)** | 2.2 ± 0.6 | 31.9 ± 2.0 | 4.1 ± 0.7 | 25.9 ± 1.2 | 23.5 ± 2.0 | 8.1 ± 0.9 |
|  | **Unfilled or unfertile grains (%)** | 5.0 ± 0.6 | 32.5 ± 1.6 | 13.3 ± 1.6 | 35.1 ± 1.6 | 48.0 ± 3.5 | 9.2 ± 1.4 |
| **Pollination type** | | Chasmogamy | Cleistogamy | Chasmogamy | Cleistogamy | Cleistogamy | Chasmogamy |

Means ± SE are shown. The numbers in parentheses (blue) indicate the percentage ratio, calculated by dividing each mean value by the corresponding mean value of WT. Length, width and thickness of grains were determined from 50-126 completely filled grains. Plant height and grain production were determined from 28-55 and 20-30 rice plants at the mature stage, respectively.

**Supplemental Table 5.** STS markers used for mapping

| ***Marker name*** | ***Sequences (5'-3')*** | | ***Product size (bp)*** | ***BAC*** |
| --- | --- | --- | --- | --- |
|  | **Forward** | **Reverse** |  |  |
| a6 | ACCGAAGAAGATGGAACTGACG | ACCTGAGAAACGAACGAAGCCTAAG | 290/276 | AC093492 |
| a9 | ATCCAACGACCGATGCTTTCC | TCTGATACAGCGGGTTTCCAC | 291/274 | AC087551 |
| a13 | ACAACCTCCACTCCCACAAG | CAAGAACACCGAGCCACGAA | 119/131 | AC087551 |
| a19 | CACAGCCATTTAGTGCGAA | CCTATGTTATCTACAGCCT | 253/267 | AC087551 |
| a32 | ACCTGAATTGGAGGGAGT | TCCTTCCATTTACGGTTG | 518/490 | AC087551 |
| a45 | CCATCCGATTTTAGGTTC | CCATTCCCACTTTCTCAT | 204/191 | AC093492 |
| a66 | TGCTTATGGTCAAACAGG | TCAGGCAATACTTTACACAC | 133/126 | AC087551 |

**Supplemental Movies**

**Supplemental Movie 1-4.** Time-lapse movies of WT (Supplemental Movie 1 and 3) and *sar1* (Supplemental Movie 2 and 4) root cells expressing EGFP-tagged α-tubulin.

Blue arrowheads indicate the position of selected microtubules. The Red arrowheads track the plus ends of the selected microtubules. White numbers indicate the elapsed time (minutes: seconds). Scale bar=5 µm.

**Supplemental Methods**

**Phenotypic Analyses**

Grains or seeds collected from primary panicles were dried for 2 days at 50°C and their awns were removed prior to measurements. The length, width, and thickness of mature florets, fully filled grains, and caryopses (dehulled grains without glumes) were measured with a digital vernier caliper. The weight of grains or brown rice grains was measured in grams as the average weight of three different samples of 100 fully filled grains and converted to 1000-grain weight for easy comparisons with previous studies. The length and width of developing florets were measured under a stereo microscope with a calibrated ocular accurate 0.01 mm

After the completion of flowering, plant height was measured from the base of the plant to the top of the main culm. Internode lengths between nodes were measured using the main culms after the removal of leaves.

To measure the length of crown roots, seeds were germinated on filter paper saturated with distilled water for 24 hours at 30°C and transferred to pots containing the half-strength Kimura B nutrient solution [0.18 mM (NH_4_)_2_SO_4_, 0.27 mM MgSO_4_, 0.09 mM KNO_3_, 0.09 mM KH_2_PO_4_, 0.05 mM K_2_SO_4_, 0.18 mM Ca(NO_3_)_2_, 0.04 mM NaEDTA-Fe, 0.08 mM Na_2_SiO_3_, pH5.5]. Before measurement of root length, germinated seedlings were grown for 7-28 days under 14 h/30°C light and 10 h/26 °C dark cycles. The liquid media were renewed every 3 days.

For observation of vascular bundles in lodicules, open and unopened florets were fixed in carnoy's solution (acetic acid: ethanol=1:3) immediately after collection. Before observation, lodicules were dissected out from the florets and stained with aceto carmine (1% carmine, 45% acetic acid).

**Positional Mapping**

The *sar1* mutants were isolated after ^60^Co γ-ray irradiation of the *japonica* cultivar Zhonghua 11 grains. An F_2_ mapping population was generated from a cross between the *sar1* mutant and the *Indica* cultivar Nanjing 11. For gene mapping, plants producing small and round grains were selected from the F_2_ population and their leaves were used to extract genomic DNA by the CTAB method. To fine map the mutant gene, more STS markers (their primer sequences are listed in Supplemental Table 5) were developed based on the sequence differences between *Indica* variety 9311 and *Japonica* variety Nipponbare (http://www.gramene.org/resources/). PCR products were separated by electrophoresis on 3.0% (w/v) agarose gels; then linkage analysis was performed between the mutant locus and molecular markers.

**Complementation and RNA Interference**

To construct the overexpression plasmid pUN1301-OsKINESIN-13A for complementation test, the full-length OsKINESIN-13A cDNA was first synthesized from total panicle (20 cm in length) RNA by use of the primer pairs PF1 (5'-ATAAGGATCCATGGGGGACTCC GGGGAC-3', *Bam*H I) and PR1 (5'-ACCGG AGCTCTTATCTGGAAGATTTCTT-3', *Sac* I), then digested with the corresponding restriction enzymes and inserted into the binary plasmid pUN1301 ([Wang et al., 2004](#_heading=h.4i7ojhp)), which was modified from pCAMBIA-1301 (NCBI accession number: AF234297) by adding the maize Ubi-1 promoter ([Christensen et al., 1992](#_heading=h.1t3h5sf)) between *Hin*d III and *Bam*H I sites and the NOS terminator between *Sac* I and *Eco*R I sites.

For RNAi construct, A 609-bp cDNA fragment (nucleotides 1587 to 2195) unique to *OsKINESIN-13A* was amplified by RT-PCR with the primer pairs PF2 (5'-GGGGTACCACTAGTTGACAGGGTTAAAAGTCTC-3', *Kpn* I and *Spe* I) and PR2 (5'-GGGGATCCGAGCTCATTTCCACATCATCACAAG-3', *Bam*H I and *Sac* I). The amplified RNAi fragments were double digested with *Spe* I / *Sac* I and *Kpn* I / *Bam*H I respectively, and then inserted in the sense and antisense direction respectively, into the binary RNAi tool vector pTCK303 ([Wang et al., 2004](#_heading=h.4i7ojhp)), derived from pUN1301, to generate the RNAi plasmid pOsKINESIN-13A-RNAi.

The constructs described above and their controls were transferred into an *Agrobacterium tumefaciens* strain EHA105 by a freeze-thaw method ([Hofgen and Willmitzer, 1988](#_heading=h.17dp8vu)). The resulting strain carrying the pUN1301 control or the pUN1301-OsKINESIN-13A construct was used to transform rice calli induced from mature embryos of *sar1*; while the strain harboring the pOsKINESIN-13A-RNAi plasmids were used to transform embryogenic calli of WT. Transformed calli were selected and induced to generate transgenic T0 plants as previously described ([Deng and Wang, 2007](#_heading=h.4d34og8)). Seeds harvested from T0 plants were selected on 1/2 MS medium containing 40 mg/L of hygromycin B. The surviving T1 plantlets were transferred to soil and grown for phenotypic analysis.

**Phylogenetic Analysis**

To identify plant members of kinesin-13 family in genome sequences recently available at NCBI, BLASTP searches were conducted against the non-redundant database by using all three motor domain sequences of the OsKinesin-13A, AtKinesin-13A, and mouse Kif2 as queries. Retrieved kinesin-13 sequences were extracted from the database and analyzed by SMART ([Letunic et al., 2009](#_heading=h.1ksv4uv)) to obtain motor domain sequences.

Alignment of all obtained amino acid kinesin-motor-domain sequences was performed using clustalw2 ([Larkin et al., 2007](#_heading=h.35nkun2)) with the default settings. Phylogenetic tree was built from the sequence alignment by PAUP v. 4b10 ([Swofford, 2003](#_heading=h.1y810tw)) using the parsimony method described previously ([Dagenbach and Endow, 2004](#_heading=h.1t3h5sf)). To provide confidence estimates, bootstrap analysis was conducted using 1000 replicates and the heuristic search algorithm with random stepwise addition, TBR swapping and maximum parsimony settings. The final tree was arbitrarily rooted using the orphan yeast kinesin ScSmy1 as an outgroup ([Kim and Endow, 2000](#_heading=h.26in1rg)).

**Morphology observation**

For observations on the development of caryopses, florets located on the upper primary rachis branches ([Ishimaru et al., 2003](#_heading=h.3rdcrjn)) were labeled on the day of flowering (day 0). In detail, WT florets with open glumes and *sar1* florets with elongated anther-filaments were labeled on the glumes at the time of flowering (between 11 a.m. and 2 p.m.). The labeled florets or grains were harvested for observation 0, 1, 3, 4, 6, 8, 12, 16 and 20 days after flowering.

Glume-cutting experiments were performed on the day of anthesis. WT and *sar1* florets were marked as described above. The distal ends of the marked glumes were cut off after the finish of flowering (at 6 p.m.), ensuring the remnant parts of glumes had the same length. The glume-cutting grains were covered by paper bags and allowed to develop until maturity. Mature caryopses that developed under glume-cutting conditions were measured with a digital vernier caliper.

**Expression and Purification of Recombinant Proteins**

To obtain recombinant motor domain of OsKinesin-13A (amino acids 170-552), the cDNA fragment of *OsKINESIN-13A* was PCR amplified using the primer pairs PF3 (5’-TTAGAATTCCTTCCAGGAAGCCAGCCAG-3’) and PR3 (5’-ACACTCGAGAGGTATAGTTGGCCCAGT-3’), and cloned in-frame into the polyhistidine (His)-tagged expression vector pET-28a (+) (Novagen) at *Eco*R I and *Xho* I sites. The construct was confirmed by sequencing (Life Technologies) and transformed into *Escherichia coli* (*E. coli*) strain BL21 (DE3). After 1 mM isopropyl-β-D-thiogalactopyranoside (IPTG) induction for 5 h at 30°C, the His-tagged OsKinesin-13A-motor protein was purified on Ni-NTA agarose columns (QIAGEN) under non-denaturing conditions according to the manufacturer's manual. The eluted OsKinesin-13A-motor protein was dialyzed overnight at 4°C against a PN buffer (15 mM PIPES-NaOH, 150 mM NaCl, PH 7.0) and concentrated by ultrafiltration with an Amicon Ultra-15 centrifugal filter (10 kDa MWCO; Millipore). Protein concentrations were determined by the Bradford assay using BSA as a standard.

To obtain recombinant full-length OsKinesin-13A proteins, the coding region of *OsKINESIN-13A* was PCR amplified using the forward primer PF4 (5'- TTAGGATCCATGGGGGACTCCGGGGAC-3') and the reverse primer PR4 (5'- GCGCTCGAGTTATCTGGAAGATTTCTTACG-3'). PCR fragments were then inserted in-frame into the GST expression vector pGEX-4T-1 (GE Healthcare Bio-Sciences) at *Bam*H I and *Xho* I sites to generate the recombinant plasmid pGEX-OsKINESIN-13A. The pGEX-4T-1 (as controls) and pGEX-OsKINESIN-13A plasmids were respectively transformed into *E. coli* strain BL21 (DE3) after confirmation by DNA sequencing (Life Technologies). The transformed *E. coli* BL21 cells were induced with 0.1 mM IPTG for 5 h at 25°C and then collected by centrifugation at 5,000 g. All collected cells were immediately frozen in liquid nitrogen, and stored at -80°C. The frozen cell pellets were resuspended in ice-cold lysis buffer [140 mM NaCl, 2.7 mM KCl, 10 mM Na_2_HPO_4_, 1.8 mM KH_2_PO_4_, 1 mg/ml lysozyme, 2 mM DTT, 1 mM PMSF, 1:50 protease inhibitor cocktail (Roche Applied Science), 1 mM MgCl_2_, 1 mM ATP, 0.1% Triton X -100, pH 7.5] and broken by sonication. Cell lysates were clarified at 15,000 g for 20 min, and the supernatants were purified using Glutathione Sepharose 4B beads (GE Healthcare Bio-Sciences) according to the user manual. Beads bound to GST (as controls) or GST-OsKinesin-13A fusion proteins were directly used for tubulin binding assay or subjected to further purification. To remove GST tag, the sepharose-bound GST-OsKinesin-13A proteins were washed with PEM buffer (100 mM PIPES, 1 mM EGTA, 1 mM MgCl_2_, 2 mM ATP, 2 mM DTT, pH 6.9), followed by overnight on-column cleavage with thrombin (GE Healthcare Bio-Sciences) at 4°C. The eluted full-length OsKinesin-13A protein were dialyzed overnight at 4°C against PKEM buffer (20 mM PIPES-KOH, 150 mM KCl, 1 mM EGTA, 1 mM MgCl_2_, 0.1 mM DTT, pH 6.9), and used for microtubule depolymerization assays.

For antigen production, a 5' coding region of *OsKINESIN-13A* (nucleotides 69 to 489) was PCR amplified using the primer pairs PF5 (5’-TATGAATTCTCCTCCACCTCCTCCTCCT-3’) and PR5 (5’-ACACTCGAGATCATCCTCATCCATTTCC-3’) and cloned into pET-28a (+) at *Eco*R I and *Xho* I sites. After sequencing confirmation, the recombinant N-terminal peptide of OsKinesin-13A (His-N-OsKinesin-13A) was expressed and purified using the method described in the purification of OsKinesin-13A-motor.

**ATPase Assay**

To detect the ATPase activity of OsKinesin-13A, the Enzyme Linked Inorganic Phosphate Assay (ELIPA) were used to measure nanomole quantities of inorganic phosphate (Pi), which probes the kinetics of Pi release from ATPases. The ELIPA reactions were performed with a Kinesin ELIPA biochem kit (Cytoskeleton Inc.) according to the manufacturer’s instructions. Briefly, the purified OsKinesin-13A-motor protein (400 nM) was incubated with or without microtubules (0.66 μM) in 600 μL reaction buffer (0.2 mM MESG, 0.3 U PNP, 15 μM paclitaxel, 15 mM PIPES, 5 mM MgCl_2_, pH 7). After adding 0.5 mM ATP (Sigma) to start the reaction, changes in absorbance was measured at 360 nm every 30 seconds over a 20-minute period, by using a DU730 Spectrophotometer (Beckman). Absorbance values were converted to nanomole quantities of Pi according to the Pi standard curve generated with Pi standards. To test whether the ATPase activity of OsKinesin-13A-motor is concentration-dependent, various amounts of OsKinesin-13A-motor (866, 433, 216, 108 nM motor) were used. Control reactions were carried out in the absence of OsKinesin-13A-motor or ATP.

**Antibody Production and Specificity Confirmation**

To produce antibodies against OsKinesin-13A, approximately 3 mg of purified His-N-OsKinesin-13A peptide was used to immunize rabbits. After three immune injections, polyclonal rabbit antisera were collected and stored at -80°C. To ensure antibody specificity, the stored antisera were affinity-purified with the immunizing peptide using the following procedure. About 100 μg of purified His-N-OsKinesin-13A proteins were electrophoresed in a 12.5% SDS-PAGE gel and then electrically transferred onto a polyvinylidene difluoride (PVDF) membrane. After blocking with 3% bovine serum albumin (BSA) in PBST buffer (140 mM NaCl, 2.7 mM KCl, 10 mM Na_2_HPO_4_, 1.8 mM KH_2_PO_4_, 0.1% Triton X -100, pH 7.5) for 1 hour, the membrane piece carrying the band of His-N-OsKinesin-13A was cut out and incubated overnight at 4°C with 14 ml of anti-sera (in PBST) at a dilution ratio of 7. The membrane piece was rinsed thoroughly with PBST, followed by vigorously washing with 1 ml of 100 mM Glycine-HCl (pH 2.7) to detach antibodies from the membrane. The antibody solution was immediately mixed with 200 μl of 1M Tris-HCl (pH 7.5) and stored at 4°C until immunostaining.

For confirmation of the antibody specificity by Western blot analysis, total proteins were extracted from 6-day-old seedlings of wild-type and *sar1* with a TM buffer (100 mM Tris-HCl, 10 mM MgCl_2_, 18% sucrose, 2% SDS, pH 8.0) supplemented with 1 mM DTT and 1 mM PMSF as described ([Nelson, 1984](#_heading=h.z337ya)). Protein concentrations were determined using Pierce™ BCA Protein Assay Kit (Thermo). The extracted supernatant proteins were separated by SDS-PAGE (12.5% gels) and blotted onto the PVDF membrane. After blocking with 5% low-fat milk powder in PBST, the membrane was probed first with the affinity-purified rabbit anti-OsKinesin-13A antibody (1/2000 diluted) and subsequently with a horseradish peroxidase conjugated secondary antibody. Hybridized signals were detected by chemiluminescence using a FluorChem FC2 imaging system (Cell Biosciences).

**Immunogold electron microscopy**

Briefly, root tips from 3-day-old rice seedling were fixed in PME containing 3% (w/v) paraformaldehyde and 2.5% (v/v) glutaraldehyde for 2 hours at room temperature and 2 hours at 4°C, followed by postfixing in 1% (w/v) Osmium tetroxide for 1 hour. After washing in PME, the fixed root tips were dehydrated in a graded alcohol series and embedded in LR White acrylic resin (Sigma). The embedded root tips were sectioned longitudinally at 60 nm and mounted on nickel grids. After treatment with 5% (w/v) sodium metaperiodate for 20 min, the root sections on grids were hybridized with the 1/50 diluted rabbit anti-OsKinesin-13A antibody and then with goat anti-rabbit IgG conjugated to 10-nm colloidal gold (Sigma). Prior to observation with a JEM-1230 transmission electron microscopy (JEOL), the sections were counterstained with 2% (w/v) uranyl acetate, washed with water and dried at room temperature.

**Preparation of microsomal membrane and fractions**

Crude microsomal membrane and fractions were prepared according to the described method ([Pertl et al., 2009](#_heading=h.3j2qqm3)). Briefly, 1.5 g leaves from two-week-old WT seedlings were cut into small pieces and homogenized on ice with a Potter-Elvehjem homogenizer (Wheaton) in 15 mL extraction buffer (330 mM sucrose, 150 mM KCl, 1 mM EDTA, 50 mM Tris, the pH was adjusted to 7.5 with MES) supplemented with 1×protease inhibitor cocktail (Roche). The homogenate was passed through a 100-μm nylon filter (Millipore), and then centrifuged at 1,500 g for 10 min and 10,000 g for 20 min at 4°C. The supernatant was centrifuged at 100,000g for 2 h at 4°C, and the pellet (crude microsomal membrane) was resuspended in ice-cold resuspension buffer (330 mM sucrose, 25 mM Tris/MES, pH 7.0) containing 1×protease inhibitor cocktail. The crude microsomal membrane (120-150 μL) was loaded onto a discontinuous sucrose gradient [1mL 18% (w/w), 1 mL 25% (w/w), 1 mL 30% (w/w), 1 mL 34% (w/w), 0.75 mL 38% (w/w), 0.25 mL 45% (w/w) sucrose prepared in precooled 1 mM MgSO_4_, 1 mM Tris/MES, pH 7.2] and centrifuged at 150,000 g (37,400 rpm, MLS-50 rotor, Beckman) for 1 h 20 min at 4°C. The resulting fractions (0.6 mL/fraction) were carefully collected from top to bottom, mixed with 5×SDS loading buffer and boiled for 4 min. Equal volume of fractions (20 μL) were separated on 12% SDS-PAGE and subjected to Western blot analysis using the anti-OsKinesin-13A antibody or rabbit polyclonal antibodies (Agrisera) against marker proteins for the plasma membrane (H^+^-ATPase), endoplasmic reticulum (SMT1), Golgi apparatus (Arf1), COPI-coated vesicles (Sec21p) and COPII-coated vesicles (Sar1).

**Supplemental References**

**Christensen, A.H., Sharrock, R.A., and Quail, P.H.** (1992). Maize polyubiquitin genes: structure, thermal perturbation of expression and transcript splicing, and promoter activity following transfer to protoplasts by electroporation. Plant Mol. Biol. **18,** 675-689.

**Dagenbach, E.M., and Endow, S.A.** (2004). A new kinesin tree. J. Cell Sci. **117,** 3-7.

**Deng, Z.-Y., and Wang, T.** (2007). *OsDMC1* is required for homologous pairing in *Oryza sativa*. Plant Mol. Biol. **65,** 31-42.

**Guo, L., Ho, C.-M.K., Kong, Z., Lee, Y.-R.J., Qian, Q., and Liu, B.** (2009). Evaluating the microtubule cytoskeleton and its interacting proteins in monocots by mining the rice genome. Ann. Bot. **103,** 387-402.

**Hofgen, R., and Willmitzer, L.** (1988). Storage of competent cells for Agrobacterium transformation. Nucl Acids Res **16,** 9877.

**Ishimaru, T., Matsuda, T., Ohsugi, R., and Yamagishi, T.** (2003). Morphological development of rice caryopses located at the different positions in a panicle from early to middle stage of grain filling. Funct. Plant Biol. **30,** 1139-1149.

**Kim, A., and Endow, S.** (2000). A kinesin family tree. J. Cell Sci. **113,** 3681-3682.

**Kitagawa, K., Kurinami, S., Oki, K., Abe, Y., Ando, T., Kono, I., Yano, M., Kitano, H., and Iwasaki, Y.** (2010). A novel kinesin 13 protein regulating rice seed length. Plant Cell Physiol. **51,** 1315-1329.

**Larkin, M.A., Blackshields, G., Brown, N.P., Chenna, R., McGettigan, P.A., McWilliam, H., Valentin, F., Wallace, I.M., Wilm, A., Lopez, R., Thompson, J.D., Gibson, T.J., and Higgins, D.G.** (2007). Clustal W and Clustal X version 2.0. Bioinformatics **23,** 2947-2948.

**Letunic, I., Doerks, T., and Bork, P.** (2009). SMART 6: recent updates and new developments. Nucl Acids Res **37,** D229-232.

**Li, M., Zang, X., Liu, J., Yu, L., and Cai, D.** (2005). Comparison of Lodicule Structure between Cleistogamous Rice CL01 and Anthesis Rice T168 (in Chinese with English abstract). J. Huazhong Agric. Univ. **24,** 564-569.

**Maeng, J.-Y., Won, Y.-J., Piao, R., Cho, Y.-I., Jiang, W., Chin, J.-H., and Koh, H.-J.** (2006). Molecular mapping of a gene ‘ld(t)’ controlling cleistogamy in rice. Theor Appl Genet **112,** 1429-1433.

**Nelson, T.** (1984). Light-regulated gene expression during maize leaf development. J Cell Biol **98,** 558-564.

**Pertl, H., Schulze, W.X., and Obermeyer, G.** (2009). The Pollen Organelle Membrane Proteome Reveals Highly Spatial−Temporal Dynamics during Germination and Tube Growth of Lily Pollen. J. Proteome Res. **8,** 5142-5152.

**Swofford, D.** (2003). PAUP*: Phylogenetic Analysis Using Parsimony (*and other methods). (Sunderland, Massachusetts: Sinauer Associates ).

**Wang, Z., Chen, C., Xu, Y., Jiang, R., Han, Y., Xu, Z., and Chong, K.** (2004). A practical vector for efficient knockdown of gene expression in rice ( *Oryza sativa* L.). Plant Mol. Biol. Report. **22,** 409-417.

**Yoshida, H., Itoh, J.-I., Ohmori, S., Miyoshi, K., Horigome, A., Uchida, E., Kimizu, M., Matsumura, Y., Kusaba, M., Satoh, H., and Nagato, Y.** (2007). superwoman1-cleistogamy, a hopeful allele for gene containment in GM rice. Plant Biotechnol. J. **5,** 835-846.
